# Sustained glymphatic transport and impaired drainage to the nasal cavity observed in multiciliated cell ciliopathies with hydrocephalus

**DOI:** 10.1186/s12987-022-00319-x

**Published:** 2022-03-05

**Authors:** Yuechuan Xue, Zachary Gursky, Brittany Monte, Sunil Koundal, Xiaodan Liu, Hedok Lee, Tatyana V. Michurina, Kennelia A. Mellanson, Lucy Zhao, Alice Nemajerova, Kristopher T. Kahle, Ken-Ichi Takemaru, Grigori Enikolopov, Natalia I. Peunova, Helene Benveniste

**Affiliations:** 1 Department of Anesthesiology, Yale School of Medicine, New Haven, CT USA; 2 Department of Critical Care Medicine, Peking Union Medical College Hospital, Peking Union Medical College, Chinese Academy of Medical Science, Beijing, China; 3 Department of Anesthesiology, Renaissance School of Medicine, Stony Brook, NY USA; 4 Center for Developmental Genetics, Stony Brook University, Stony Brook, NY USA; 5 Department of Pathology, Renaissance School of Medicine, Stony Brook, NY USA; 6 Division of Pediatric Neurosurgery, Massachusetts General Hospital, Boston, MA USA; 7 Department of Pharmacological Sciences, Renaissance School of Medicine, Stony Brook, NY USA; 8 Department of Biomedical Engineering, Yale School of Medicine, New Haven, CT USA; 9 Present Address: Keck School of Medicine, University of Southern California, Los Angeles, CA USA

**Keywords:** Ciliopathy, Multiciliated cell, Hydrocephalus, CEP164, p73, Mouse model, Glymphatic, Waste drainage, CNS fluid homeostasis

## Abstract

**Background:**

Hydrocephalus (increased ventricular size due to CSF accumulation) is a common finding in human ciliopathies and in mouse models with genetic depletion of the multiciliated cell (MCC) cilia machinery. However, the contribution of MCC to CSF dynamics and, the mechanism by which impaired MCC function leads to hydrocephalus remains poorly understood. The aim of our study was to examine if defects in MCC ciliogenesis and cilia-generated CSF flow impact central nervous system (CNS) fluid homeostasis including glymphatic transport and solute waste drainage.

**Methods:**

We used two distinct mouse models of MCC ciliopathy: MCC-specific CEP164 conditional knockout mice (FOXJ1-Cre;CEP164^fl/fl^ (N = 10), 3-month-old) and p73 knock-out (p73^−/−^ (N = 8), 5-month-old) mice. Age-matched, wild-type littermates for each of the mutants served as controls. Glymphatic transport and solute drainage was quantified using in vivo T1 mapping by magnetic resonance imaging (MRI) after CSF infusion of gadoteric acid. Brain morphometry and aquaporin 4 expression (AQP4) was also assessed. Intracranial pressure (ICP) was measured in separate cohorts.

**Results:**

In both of the two models of MCC ciliopathy we found the ventriculomegaly to be associated with normal ICP. We showed that FOXJ1-Cre;CEP164^fl/fl^ mice with hydrocephalus still demonstrated sustained glymphatic transport and normal AQP4 expression along capillaries. In p73^−/−^ mice glymphatic transport was even increased, and this was paralleled by an increase in AQP4 polarization around capillaries. Further, solute drainage via the cribriform plate to the nasal cavity was severely impaired in both ciliopathy models and associated with chronic rhinitis and olfactory bulb hypoplasia.

**Conclusions:**

The combination of sustained glymphatic transport, impaired solute drainage via the cribriform plate to the nasal cavity and hydrocephalus has not previously been reported in models of MCC ciliopathy. Our data enhance our understanding of how different types of ciliopathies contribute to disruption of CNS fluid homeostasis, manifested in pathologies such as hydrocephalus.

**Supplementary Information:**

The online version contains supplementary material available at 10.1186/s12987-022-00319-x.

## Introduction

There has been a growing and evolving interest in the functional and biochemical drivers of fluid homeostasis in the central nervous system (CNS) [1, 2]. Recent studies have shown that circulation of cerebrospinal fluid (CSF) through the glymphatic system, along the cranial nerves including the olfactory nerves running through the cribriform plate as well as meningeal lymphatics facilitates waste disposal and immune surveillance of the CNS [3–5]. CSF circulation is required for waste disposal via the glymphatic system and disruptions such as CSF leaks from a dural tear or the arrest of CSF production reduces glymphatic function [6]. While aging is commonly linked to cognitive dysfunction, aging is also associated with a decline in CSF production [7] and a decrease in glymphatic–lymphatic system function [8–10]. Hence, therapeutic efforts directed towards maintaining CSF circulation and CNS fluid homeostasis across the given life span may be beneficial for preserving cognitive health.

Non-invasive magnetic resonance imaging (MRI) studies have shown that CSF moves from its major site of production—the choroid plexuses of the ventricles—through the serially connected 3rd, and 4th ventricles into the subarachnoid space [11, 12]. From the subarachnoid space, a portion of CSF is transported into the peri-vascular channels of the glymphatic system where it exchanges with interstitial fluid (ISF) and promotes waste disposal via meningeal and other extracranial lymphatics [13, 14]. CSF circulation through the CNS is driven by respiration [11] in sync with negative thoracic pressure [15], vascular pulsatility [16] and vasomotion [17]. Another contributor to CSF movement within the CNS involves the motile multi-ciliated cells (MCCs) lining the cerebral ventricles [18, 19]. Ex situ studies using organotypic cerebral ventricle explant cultures from rodents and pigs [19], as well as in vivo experiments in Xenopus [20, 21] and zebrafish [22], have revealed intricate dynamic patterns of CSF flow through the ventricles orchestrated by the MCCs. However, the role of MCCs in CSF transport and CNS fluid regulation is incompletely understood [23]. The impact of MCCs on CSF transport has been inferred from the human condition of primary ciliary dyskinesia (PCD) and genetic rodent models of PCD [24]. PCD and other ciliopathies are often associated with communicating hydrocephalus [23, 25]. Recently, a more severe PCD phenotype in humans was reported, known as ‘reduced generation of multiple motile cilia’ (RGMC) involving mutations in the multicilin gene which is linked to a high incidence of hydrocephalus and choroid plexus hyperplasia [26, 27]. Notably, studies have shown that the CSF stroke volume and the oscillatory shear stress in the cerebral aqueduct increase in normal pressure hydrocephalus (NPH) which impede normal cilia beating [28–30]. Furthermore, a study using dynamic contrast enhanced MRI (DCE-MRI) with CSF administration of a Gd-based tracer showed that glymphatic clearance was reduced in human patients with NPH [31, 32] suggesting a potential link between impaired glymphatic clearance, cilia dysfunction and hydrocephalus.

To further bridge the gap in knowledge of the contribution of MCC to CNS fluid homeostasis we combined T1 mapping and CSF administration of a small molecular weight (MW) Gd-based solute (gadoteric acid, ‘Gd-DOTA, MW 558 Da) to quantify glymphatic-lymphatic system transport in two different mouse models of ciliopathy. Specifically, we used (1) MCC-specific CEP164 conditional knockout mice (FOXJ1-Cre; CEP164^fl/fl^) [33] and (2) p73 knock-out (p73^−/−^) mice which lack both TAp73 and DeltaNp73 isoforms [34]. Both of these ciliopathies have normal primary cilia in non-ciliated cells [33, 35–37] but exhibit a significant loss of MCC in the airways, oviduct, and ependyma, and are also associated with hydrocephalus and other abnormalities characteristic of human ciliopathies [24, 27, 33, 34]. The aim of our study was to examine if defects in MCC ciliogenesis and cilia-generated CSF flow impact the dynamics of the glymphatic transport and solute drainage from the CNS. We hypothesized that glymphatic transport as well as solute drainage would be impaired in both mouse models with genetic mutations affecting the function of cilia of the ependymal MCCs.

## Materials and methods

### Animals

Male and female mice were bred in-house at Stony Brook University. The following strains were used: FoxJ1-Cre;CEP164^fl/fl^, CEP-164^fl/fl^, p73^+/+^ and p73^−/−^ (Table 1). FoxJ1-Cre;CEP164fl/fl mice were created as previously described [33] by crossing FOXJ1-Cre;CEP164^fl/+^ with CEP164^fl/fl^ mice. Genotyping was performed on tail biopsies using the following PCR primers: CEP164 KO-first, 5ʹ-CCATCTGTCCAGTACCATTAAAAA-3ʹ and 5ʹ-CCCAGAATACAACATGGGAGA-3ʹ (WT allele, 215 bp; floxed allele, 415 bp); Cre, 5ʹ-CGTATAGCCGAAATTGCCAGG-3ʹ and 5ʹ-CTGACCAGAGTCATCCTTAGC-3ʹ (327 bp). The p73^−/−^ mice with a deletion of exons 5 and 6 of the *TP73* gene were a generous gift from Dr. Frank McKeon (University of Houston, TX) [34]. The mice were enriched for six generations on the SV129 background. Corresponding WT mice were littermates. The strains were barrier-maintained in the mouse facility of Stony Brook University through heterozygous interbreeding. The genotype of mice was verified by PCR amplification specific for the corresponding WT and mutant alleles. All animals were genotyped twice, at the time of weaning and prior the experiments with the following PCR primers: Primer 1, 5ʹ GGG CCA TGC CTG TCT ACA AAG AA 3ʹ, Primer 2, 5ʹ CCT TCT ACA CGG ATG AGG TG 3ʹ, Primer 3, 5ʹ GAA AGC GAA GGA GCA AAG CTG 3ʹ. WT = ~ 650 bp (Primer 1 + Primer 2), Mut = ~ 400 bp (Primer 1 + Primer 3). The mice were transported from Stony Brook University to the Yale University quarantine facility where they were allowed to rest up to 1 week before experimental procedures. Mice of all strains were housed in a temperature and humidity-controlled environment with a 12 h light/dark cycle (7:00 am–7:00 pm) and were fed regular rodent chow and water ad libitum. Experiments for FoxJ1-Cre;CEP164^fl/fl^ and CEP-164^fl/fl^ mice were conducted at ~ 3 month of age and at ~ 5 month for the p73^+/+^ and p73^−/−^ mice (Table 1). Age, sex distribution and body weights across the experimental groups are listed in Table 1. All animal experiments were approved by the Institutional Animal Care and Use Committee at Yale University and Stony Brook University in accordance with the United States Public Health Service’s Policy on Humane Care and Use of Laboratory Animals.

**Table 1:** Overview of experimental groups including mortality, MRI, age, and sex of animals included in analysis

Group	Strain	Mortality	Scan related issues	Age (weeks)	Sex, % Male	MRI morphometry	MRI glymphatics	ICP recording
1	CEP164^fl/fl^ (N = 13)	N = 1	N = 2	12.8 ± 0.5	56.0	N = 10	N = 7	No
2	FOXJ1Cre; CEP164^fl/fl^ (N = 14)	N = 3	N = 1	13.3 ± 0.4	20.0	N = 10	N = 8	No
3	p73^+/+^ (N = 8)	N = 0	N = 0	19.9 ± 1.0	62.5	N = 8	N = 8	No
4	p73^−/−^ (N = 8)	N = 1	N = 1	19.0 ± 1.0	85.7	N = 7	N = 6	No
5	CEP164^fl/fl^ (N = 9)	N = 2	N/A	22.6 ± 4.2	16.7	No	No	Yes
6	FOXJ1Cre; CEP164^fl/fl^ (N = 7)	N = 2	N/A	26.6 ± 6.4	60.0	No	No	Yes
7	p73^+/+^ (N = 5)	N = 1	N/A	49.9 ± 9.3	66.7	No	No	Yes
8	p73^−/−^ (N = 6)	N = 0	N/A	34.5 ± 3.4	83.3	No	No	Yes

Age is presented as mean ± SEM

ICP: Intracranial pressure

### Infusion of Gd-DOTA into the cisterna magna

All mice were anesthetized with intraperitoneal (IP) ketamine/xylazine (KX), (ketamine 17.5 mg/ml and xylazine 2.5 mg/ml, 0.1 ml/20 g body weight) and also received glycopyrrolate (0.2 mg/kg IP). Anesthesia was maintained with KX (0.05 ml of the KX-mixture/20 g body weight) administered every 30 min via an IP catheter. The mice were breathing spontaneously in a nose cone delivering a 1:1 air:O_2_ mixture. The mouse was mounted in a stereotaxic frame and the skin shaved and cleaned. Through a midline incision, a 34G shortened needle (Hamilton, US) connected via polyurethane tubing to a 50 µl Hamilton syringe (Hamilton, US) mounted in a micro-infusion pump (Legato 130, KD Scientific, Holliston, MA, USA) was inserted into the cisterna magna (CM). 7 µl of Gd-DOTA (Guerbet LLC, Princeton, NJ, US) prepared as a 1:20 dilution in sterile 0.9% NaCl was delivered at an infusion rate of 1 µl/min. After the infusion, the needle is left in place for 2 min to avoid backflow. Subsequently, the needle was withdrawn, and the durotomy immediately sealed with cyanoacrylate glue. The skin was closed, and the anesthetized mouse was transferred to the MRI scanner.

### Intracranial pressure recording

Intracranial pressure (ICP) was measured in separate series of mice (Table 1) anesthetized with KX as described above. A pressure sensor probe (Millar’s SPR-1000 Mikro-Tip^®^ mouse pressure catheter, Texas USA) inserted into a water-filled 30G needle and sealed by Touhy Borst Adaptor to create a closed pressure system was used for the ICP measurements. The Millar probe measures pressures in the range of 0.2–8.0 mmHg. To counterbalance gravity from pulling out the liquid inside the 30G needle and creating negative pressure, thereby inducing pressure errors, the height of the water column inside the Tuohy borst did not exceed 10–13 mmH_2_O. The pressure sensor was pre-calibrated with another pressure sensor (AD instruments, US) for a range between 0 and 30 mmHg. The anesthetized mice were mounted in a stereotaxic frame and the dura covering the CM was exposed. Using the stereotaxic frame, the 30G needle connected to the pressure sensor was inserted into the CM. For recordings, the pressure sensor was connected to a PowerLab data acquisition system (AD instruments, USA) for time resolved pressure measurements. The average pressure was calculated over 1 min after the pressure readings had stabilized (~ 1 min). Physiological parameters including heart rate, respiratory and body temperature was recorded in the animals during the ICP measurements. All animals were euthanized at the conclusion of the measurement. The analog voltage readings were measured at a 1 kHz sampling rate and were calibrated to pressure readings using a calibration table (LabChart version 8, AD instruments, USA).

### MRI imaging

All MRI acquisitions were performed on a Bruker 9.4 T/16 MRI instrument with a BGA-9S-HP imaging gradient interfaced to a Bruker Advance III console and controlled by Paravision 6.1 software (Bruker Bio Spin, Billerica, MA, USA). In vivo MRI: A volume radio-frequency (RF) transmit with an inner diameter (ID) of 5.0 cm and a 10 mm surface receive coil was utilized for the in vivo MRI scans. The 3D T1 mapping technique used in this study was based on our previously described method [38]. Briefly, this technique allows for quantitative assessment of both glymphatic transport as well as drainage to the nasal cavity and to the cervical lymph nodes [38]. Glymphatic transport is measured via a T1 map acquired at ~ 1 h after initiation of Gd-DOTA CSF infusion on the bench. The MRI procedures include: (1) an anatomical localizer along three orthogonal planes, (2) a spatial inhomogeneity profile of the RF transmit (B1+) using a double angle method via the rapid acquisition with relaxation enhancement (RARE) sequence (TR = 10,000 ms, TE = 22 ms, Average = 1, RARE factor = 4, number of slices = 36, in plane resolution = 0.24 mm/pixel, slice-thickness = 0.3 mm, slice gap = 0.2 mm Flip angles = 70° and 140°); and (3) acquisition of the 3D T1 map performed using a multiple gradient echo variable flip angle steady state spoiled gradient recalled echo (VFA-SPGR) method (TR = 16 ms, TE = 3 ms, Average = 1, scanning time = 2 min 40s, matrix = 100 × 100 × 100 reconstructed at 0.18 × 0.18 × 0.18 mm). A set of six flip angles (2°, 5°, 10°, 15°, 20°, 30°) were acquired for post-contrast T1 maps and the T1 scan required ~ 16 min. Ex vivo MRI: After in vivo scanning, animals were deeply anesthetized with KX and transcardially perfused with heparinized phosphate buffered saline (PBS) followed by neutral buffered formalin (NBF) and the head was subsequently stored in the perfusion fixation fluid for at least 24 h. Prior to imaging, the ‘in-skull’ specimens were immersed in a mixture of 2 mM Gadoteric acid (Gd-DOTA Guerbet LLC, Princeton, NJ, USA) and a phosphate buffer solution for 24 h [39] and ex vivo imaging was performed on the 9.4 T Bruker magnet using a custom RF volume transmit and receive volume coil (I.D. 1.4 cm). The specimen was placed inside a custom-made 3D printed MRI-compatible holder containing proton signal-free susceptibility matched fluid (Galden Heat Transfer Fluids, HT230. Kurt J. Lesker, Company, USA) and maintained at a room temperature (20 ± 2 °C) during the scan. A 3D T1-weighted FLASH imaging protocol was implemented using following parameters: TR = 30 ms; TE = 8 ms; NA = 2; isotropic voxel resolution of 60 µm × 60 µm × 60 µm requiring a total acquisition time of 43 min per specimen.

### MRI imaging processing and analyses

The 3D T1 maps were calculated using linearization of the SPGR signals and unweighted least square fit as described previously [38, 40]. In house software was written in Matlab 2017 (Mathworks, MA, USA) and used for all processing unless otherwise specified. Glymphatic transport analysis was performed via T1 cluster analysis as previously described [38]. Briefly, glymphatic transport was measured by selecting voxels with a T1 in the range of 1–1700 ms from the brain parenchymal T1 maps and converting into a 3D volume rendered binary map using PMOD software (PMOD—Biomedical Image Quantification Software Version 4.205 (c) 1996–2021 by PMOD Technologies Ltd, Zurich, Switzerland). For a given mouse the volume of voxels defined as 1 ms ≤ T1 ≤ 1700 ms represents glymphatic transport of Gd-DOTA over the given circulation time of ~ 1 h. For solute drainage, the nasal cavity and deep cervical lymph nodes were anatomically outlined, and the voxels with T1 in the range of 1–1700 ms were extracted from each region of interest and converted into a volume representing Gd-DOTA drainage. Glymphatic transport, nasal cavity drainage and drainage to the deep cervical lymph nodes (dcLN) were displayed as T1 maps of voxels in the range of 1–1700 ms overlaid on the corresponding anatomical template using Amira software (Amira 6.5.0, Thermo Fisher Scientific, USA). Anatomical MRIs comprised the 3D low flip angle (2° and 5°) SPGR images which were summed to increase signal-to-noise ratios. Analyses of in vivo MRI data included morphometric analysis of the SPGR-low FA and T1 maps for quantification of total intracranial volume (TIV), brain parenchymal volume, cerebral ventricle volume and olfactory bulb volume. The right and the left dcLN were also manually outlined on the anatomical MRI images and the total dcLN volume calculated for each of the mice. These regions were manually outlined by anatomical experts (LZ, HB and SK) using PMOD software (PMOD—Biomedical Image Quantification Software Version 4.205 (c) 1996–2021 by PMOD Technologies Ltd, Zurich, Switzerland). Similarly, for the ex vivo MRI scans the hippocampal region and choroid plexuses were manually outlined and the volumes extracted from each mouse using PMOD software (PMOD Technologies Ltd, Zurich, Switzerland).

### Histological analysis of AQP4

The analysis of the perivascular AQP4 expression pattern across the groups was focused on the hippocampus. The hippocampus region was chosen for several reasons: First, the hippocampus region represents a large subcortical structure fed by perforating arterioles in the rat brain which is included in the ‘glymphatic transport’ metric captured by the T1 mapping method. Second, we have previous experience quantifying AQP4 expression patterns in this particular region [41]. Third, the ex vivo MRI data revealed unique hippocampal dysgenesis in the ciliopathy mouse strains—in particularly in the p73^−/−^ mice—which alter glymphatic transport and also the expression of AQP4. To quantify AQP4 expression associated with the vasculature in the ventral hippocampus, a combination of manual and automated methods was used on tissue sections immunofluorescently labeled for AQP4. 

#### Tissue preparation

The perfusion fixed brain samples were collected and placed in NBF for 24 h at 4 °C. The following day, samples were cryoprotected in 10%, 20% and 30% sucrose in PBS supplemented with 0.01% sodium azide. Samples were embedded in OCT, snap-frozen in isopentane super cooled by liquid nitrogen, sectioned coronally at 10 µm thickness and slide-mounted (Histoserv, Inc). 

#### Immunofluorescent visualization of perivascular AQP4

All incubations were performed at room-temperature unless specified otherwise. One slide of the ventral hippocampus (approx. − 5.80 mm bregma) was selected from each animal and thawed overnight. Once completely dried, the slides were rehydrated in PBS, placed in a 1× citrate solution (Genemed CAT#10-0020) and microwaved for 2 min for antigen retrieval. Slides were cooled at room temperature for 30 min and washed in deionized H_2_O. Sections were then permeabilized using PBS/0.3% Triton X-100, incubated for 30 min in Image-iT FX Signal Enhancer (Thermo Fisher Scientific) and blocked for 1 h using Blockaid blocking solution/0.3% Triton X-100 (Thermo Fisher Scientific). Sections were incubated overnight at 4 °C in rabbit anti-AQP4 primary antibody (Novus Biologicals, NBP1-87679, 1:400), rinsed in PBS/0.1% Triton X-100, incubated for 2 h in donkey anti-rabbit Alexa Fluor Plus 647 (Thermo Fisher Scientific, 1:1000), cover slipped, and allowed to harden overnight before imaging. Images were acquired using a Zeiss Axio Imager.Z2 microscope with Hamamatsu ORCA-Fusion Digital CMOS Camera, X-Cite Xylis LED light source, and 20× (0.80 NA) air objective. Zen Software was used to acquire a tile scan that included the entirety of the ventral hippocampus present in selected sections (Additional file 1: Fig. S1A). These images were used to determine the polarization index of AQP4 [41] as well as the area fraction of capillaries (diameter < 10 µm) and small vessels (diameter > 10 µm). All classifier training and quantification was performed by an experimenter (ZG) blind to animal genotype.

### Automated analysis of AQP4 polarization using CellProfiler and CellProfiler Analyst

The entirety of the ventral hippocampus in each image was first contoured by hand using FIJI/ImageJ software [42], the area of hippocampus was measured, and the region was isolated from the remainder of the image (Additional file 1: Fig. S1A). Images were then run through a processing pipeline using CellProfiler 4.1.3 [43] to identify all AQP4+ vessels. To summarize, vessels were identified using a “robust background” thresholding method and an area of 32.5 µm was identified to determine the median background value for calculating the polarization index for each vessel (Additional file 1: Fig. S1A). The intensity and intensity distribution of the objects and their surrounding area were exported by the software. A Neural Network classifier in CellProfiler Analyst 3.0.2 [44] was trained by hand using 201 randomly-selected objects to classify the objects as either a capillary, a small vessel, or neither (e.g., objects that were incorrectly screened during thresholding, objects whose orientation was clearly not perpendicular to the plane of section). Classifier accuracy was 86.5% (Additional file 1: Fig. S1B). The polarization index (PI) was calculated for each object, defined as the maximum intensity value for the object divided by the median intensity value of the surrounding area (32.5 µm radially-expanded, as mentioned earlier). This definition is similar to that used in [41] with the notable advantage that the 2-dimensional nature of the analysis eliminates potential bias of the orientation of intensity sampling line. When the data from the 10 animals from the CEP164 series analyzed using Cell Profiler were compared with the manual methodology used in Koundal et al. [41], the outcomes were significantly correlated (Pearson product-moment correlation, r_8_ = 0.646, p = 0.044, Additional file 1: Fig. S1C, D) and resulted in almost identical between-groups statistical outcomes (95% CI for manual method: − 5.358 to 1.216; 95% CI for automated method: − 3.566 to 0.815). This supports the use of the automated analysis technique to examine capillaries and small vessels in the experiments. Intensity distribution properties such as the radial coefficient of variation (“radial CV”) was also calculated by the software for each object. The radial CV is a metric which captures whether or not AQP4 is upregulated uniformly along the entire perivascular circumference (represented by the astrocytic endfeet). Although data for the AQP4 polarization index and radial CV reported in this manuscript represented the mean values in a given animal, it is critical to account for these differences based on the number of per-animal observations. Thus, the validity of a mean-value approach was confirmed using a linear mixed-effects model accounting for within-animal variability [45] as each animal had a different number of capillaries and small vessels analyzed (see Additional file 2: Table S1). The AQP4 polarization index and radial CV data were also analyzed using a mixed-effects linear model to account for within-animal variability in AQP4 expression and between-animal differences in the number of vessels detected using CellProfiler. When using animal as a random effect clustering variable [45], all AQP4-related outcomes were identical to those analyzed with t-tests supporting the robustness of these analyses.

### Quantification of AQP4+ vessel area fraction

Images examined were identical to those fed into CellProfiler for the polarization analysis (i.e., isolated individual images of AQP4 labeling in ventral hippocampus), converted to JP2 format with the hippocampus region of interest using the image processing software NeuroInfo (MBF Bioscience, Williston, VT). The analysis software Stereo Investigator (MBF Bioscience, Williston, VT) was used to acquire estimates of the vascular area fraction through a systematic random sampling protocol. The area fraction fractionator probe used an area sampling fraction of ¼ (100 µm × 100 µm counting frame systematically progressed along a randomly-placed 200 µm × 200 µm grid) and probe markers spaced every 10 µm × 10 µm within each counting frame.

### Statistical analysis

Sample sizes were chosen on the basis of similar experiments previously published [38]. Neither a priori nor a post hoc power analysis was conducted to formally determine or justify sample size due to the unknown effect size of the impact of ciliopathy on the cohorts when planning the current study. All between-group analyses (between-genotype) were performed using Welch two sample t-tests. After all the analysis, the least square (marginal) mean difference (and 95% CI) of the outcomes was calculated as the effect size estimate, which would be informative in the design of a future study in which the sample size needs to be directly calculated based on a target statistical power (e.g., 80%) and significance level (e.g., 0.05) to detect a prespecified effect size. All the analyses were performed using IBM SPSS Statistics, Version 26. A p-value of less than 0.05 was chosen to indicate statistical significance and no adjustment of multiple testing was considered.

## Results

### Experimental associated physiology and mortality

Several mouse models of ciliopathy and particularly the p73^−/−^ mouse strain are known to be associated with a variety of pathophysiology including, hydrocephalus, persistent cough and a ‘runty’ phenotype [34]. First, the variable degree of hydrocephalus observed across groups might influence the overall morbidity associated with the anesthesia and surgery. Second, given the ciliopathy the associated respiratory malaise might also impact physiological parameters and potentially impact glymphatic and lymphatic functions. We recorded body weight prior to MRI and measured heart rate (HR) and respiratory rate during the MRI experiments. While no differences in body weight was observed across the CEP164^fl/fl^ and FOXJ1-Cre;CEP164^fl/fl^ mice (p-value = 0.110, Additional file 3: Table S2), the body weight of the p73^−/−^ mice was ~ 20% lower compared to the p73^+/+^ controls (p-value = 0.027, Additional file 3: Table S2). Regarding mortality, the 3 month (M) old CEP164^fl/fl^ controls (N = 10) and age-matched FOXJ1-Cre;CEP164^fl/fl^ mice (N = 10) underwent in vivo MRI scanning for brain morphometry and glymphatic-lymphatic transport measurements and one CEP164^fl/fl^ mouse and three FOXJ1-Cre;CEP164^fl/fl^ mice died during scanning (Table 1). For the other p73 groups, MRI scanning was performed successfully on all the ~ 5 M old p73^+/+^ control (N = 8) and age-matched p73^−/−^ (N = 7) (Table 1). The physiological data recorded from the ketamine–xylazine anesthetized mice during MRI scanning showed that there were no differences in HR, respiratory rate, or body temperature across the CEP164^fl/fl^ and FOXJ1-Cre;CEP164^fl/fl^ mice. However, the HR of p73^−/−^ mice was increased by ~ 15% compared to the p73^+/+^ controls (p-value 0.011, Additional file 3: Table S2). From these data we concluded that the p73^−/−^ ciliopathy mice with lower body weight appeared to be more affected by the respiratory ciliopathy (i.e., chronic cough) compared to the FOXJ1-Cre;CEP164^fl/fl^ mice. However, the overall experimental associated mortality was higher in the FOXJ1-Cre;CEP164^fl/fl^ mice which was probably related to the variable and often severe hydrocephalus observed (see below).

### FOXJ1-Cre;CEP164fl^/fl^ mice are hydrocephalic with variable but normal mean ICP

**Fig. 1:**
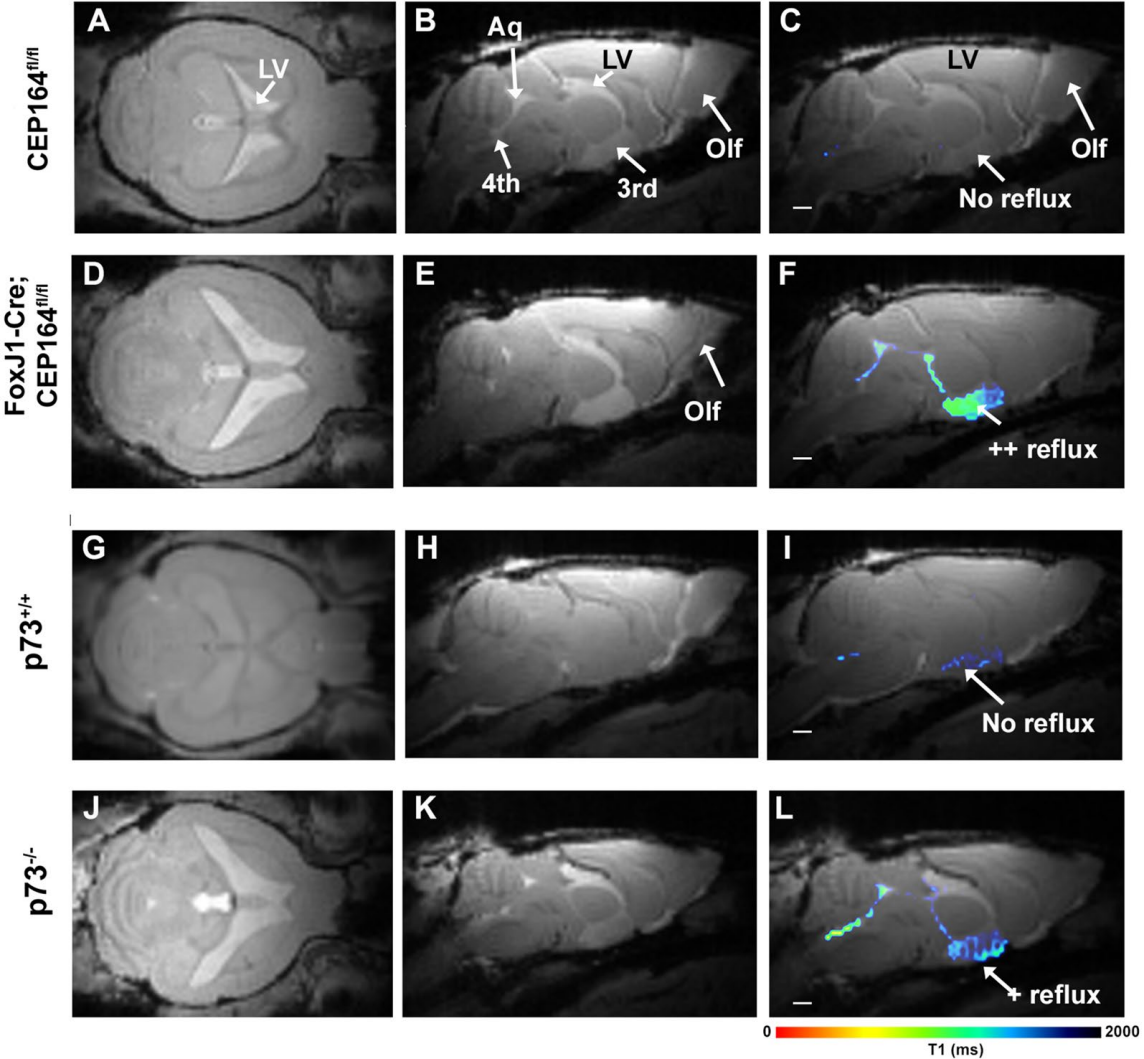
Hydrocephalus of the communicating form is present in FOXJ1-Cre;CEP164^fl/fl^ and p73^−/−^ mice. **A**, **B** Representative 2D in vivo MRI brain scans from a CEP164^fl/fl^ mice showing the fluid filled cerebral ventricles including lateral ventricles and aqueduct as areas of high signal intensity. **C** The color-coded T1 map of the CEP164^fl/fl^ control mice showing no reflux of Gd-DOTA into the cerebral ventricles overlaid onto the anatomical MRI scan. **D**, **E** 2D MRI brain scans from a FOXJ1-Cre;CEP164^fl/fl^ showing enlarged cerebral ventricles and hypoplastic olfactory bulb. **F** The T1 map from the FOXJ1-Cre;CEP164^fl/fl^ mouse shows that the Gd-DOTA tracer has refluxed into the 3rd ventricle implying abnormal CSF flow. **G**–**I** Corresponding anatomical brain scans and Gd-DOTA T1 map from a p73^+/+^ mouse. Note that the cerebral ventricles are tiny in the p73^+/+^ mouse and that there is no sign of reflux of Gd-DOTA into the 3rd ventricle. **J**–**L** Corresponding anatomical and T1 maps from a ciliopathy p73^−/−^ mouse showing enlarged cerebral ventricles, hypoplastic olfactory bulb and mild contrast tracer reflux into the 3rd ventricle. Scale bars = 1 mm. LV: Lateral ventricle; Olf: olfactory bulb; Aq: aqueduct; 3rd V: 3rd ventricle

Examination of the MRI brain scans acquired in vivo revealed expansion of the cerebral ventricles in the FOXJ1-Cre;CEP164^fl/fl^ with no obvious obstructions around the aqueduct consistent with communicating hydrocephalus when compared to the CEP164^fl/fl^ mice (Fig. 1A–E). In the FOXJ1-Cre;CEP164^fl/fl^ mice, the lateral ventricles in particular were grossly enlarged (Fig. 1D) and CSF-like fluid was often observed tracking into the parenchyma above the corpus callosum. Additional file 4: Fig. S2 shows four different FOXJ1-Cre;CEP164^fl/fl^ mice with varying degree of hydrocephalus. Notably, tracking of fluid into the parenchyma (likely from the ventricles) is also variable across the FOXJ1-Cre;CEP164^fl/fl^ mice (Additional file 4: Fig. S2). Further, the olfactory bulb was strikingly hypoplastic in the FOXJ1-Cre;CEP164^fl/fl^ mice (Fig. 1D, E) when compared to CEP164^fl/fl^ mice (Fig. 1A, B). The statistical analysis showed that the total intracranial volume (TIV) was ~ 7% larger and the olfactory bulb volume ~ 30% smaller in FOXJ1-Cre;CEP164^fl/fl^ compared to CEP164^fl/fl^ mice (Table 2). Notably, there were no differences in gross tissue volume across the two strains (p-value = 0.656, Table 2) implying that the volume of other brain regions expanded in FOXJ1-Cre;CEP164^fl/fl^ mice given the atrophy of the olfactory bulb. The presence of hydrocephalus in FOXJ1-Cre;CEP164^fl/fl^ mice was further confirmed by the fact that the cerebral ventricle volume as a fraction of TIV was ~ sevenfold larger than in CEP164^fl/fl^ mice (Table 2). In the FOXJ1-Cre;CEP164^fl/fl^ mice, Gd-DOTA tracer reflux into the 3rd ventricle was evident (Fig. 1F), signifying abnormal CSF flow dynamics.

**Table 2:** Summary of baseline in vivo brain compartment volumes across CEP164^fl/fl^ vs FOXJ1-Cre;CEP164^fl/fl^ groups

Dependent variable	CEP164^fl/fl^ (N = 10)		FOXJ1-Cre; CEP164^fl/fl^ (N = 10)		Difference	SE	p-value	L95%	U95%
	Mean	SE	Mean	SE					
Total intracranial volume, TIV (mm^3^)	478.1	5.3	512.6	11.5	− 34.5	12.6	**0.018**	− 61.9	− 7.9
Brain tissue volume (mm^3^)	468.3	4.7	471.8	6.2	− 3.5	7.7	0.656	− 19.8	12.8
Cerebral ventricle (CV) volume (mm^3^)	5.4	0.5	35.6	7.6	− 30.2	7.6	**0.003**	− 47.5	− 12.9
CV volume fraction of TIV (%)	1.1	0.1	6.7	1.2	− 5.6	1.2	**0.001**	− 8.4	− 2.9
Olfactory bulb volume (mm^3^)	20.3	0.6	13.8	0.6	6.6	0.8	**<** **0.001**	4.9	8.2

Data are presented as means and SE’s; Mean differences compare CEP164^fl/fl^ and FOXJ1-Cre;CEP164^fl/fl^ groups for each dependent variable. L95%: lower limit of 95% confidence interval for mean (CI), U95%: upper limit of 95% CI

p-values marked with bold indicate that the mean difference is significant at the 0.05 level.

To further explore the clinical phenotype of the hydrocephalic state, we measured ICP in separate series of FOXJ1-Cre;CEP164^fl/fl^ and CEP164^fl/fl^ mice (Table 1). In FOXJ1-Cre;CEP164^fl/fl^, the ICP was variable across the individual mice, but the average ICP was not significantly different when compared to CEP164^fl/fl^ control mice (FOXJ1-Cre;CEP164^fl/fl^ (N = 5) mean ICP = 6.8 ± 2.3 mmHg vs CEP164^fl/fl^ (N = 7) mean ICP = 4.0 ± 1.0 mmHg, mean difference = − 2.5 mmHg, 95% CI [− 5.7, − 0.1] mmHg, p-value = 0.055). Given the combination of normal mean ICP in the FOXJ1-Cre;CEP164^fl/fl^ mice, we designated the hydrocephalic state as normal pressure communicating hydrocephalus.

Hippocampal dysgenesis has previously been reported in ciliopathy mouse models [34]. We performed higher spatial resolution ex vivo MRI scans—voxel resolution of 60 µm^3^—to explore and quantify hippocampal morphometry. Anatomical ex vivo MRI brain scans showed that the CA1 and CA3 pyramidal cell layers of the hippocampus, as well as the granule cell layer of the dentate gyrus, were visible as bright signal intensity bands in both CEP164^fl/fl^ and FOXJ1-Cre;CEP164^fl/fl^ mice (Additional file 5: Fig. S3A, C, E, G). In the CEP164^fl/fl^ mouse hippocampal anatomy appeared normal (Additional file 5: Fig. S3A, E) whereas in the FOXJ1-Cre;CEP164^fl/fl^ mouse the upper, supra-pyramidal blade of the granule cell layer of the dentate gyrus appeared with a sharp ‘bend’ (white arrow, Additional file 5: Fig. S3C, G). The volume of the whole hippocampus was ~ 15% larger in the FOXJ1-Cre;CEP164^fl/fl^ compared to controls: (FOXJ1-Cre;CEP164^fl/fl^ (N = 7) hippocampus volume = 30.0 ± 3.5 mm^3^ vs CEP164^fl/fl^ controls (N = 7) hippocampus volume = 25.8 ± 0.3 mm^3^, mean difference = − 4.2 mm^3^, 95% CI [− 7.3, − 1.0] mm^3^, p-value = 0.016). On the ex vivo scans the curly loops of the choroid plexus were clearly visible within the enlarged lateral ventricles of the FOXJ1-Cre;CEP164^fl/fl^ mice (red arrows, Additional file 5: Fig. S3C, D, H). The choroid plexus volume of the FOXJ1-Cre;CEP164^fl/fl^ mice (N = 7) was 0.49 ± 0.08 mm^3^, which would translate into ~ 0.5 mg. In the CEP164^fl/fl^ controls the choroid plexus could not be quantified due to the ventricles collapsing in the post-mortem specimens (Additional file 5: Fig. S3F).

### p73^−/−^ ciliopathy mice are hydrocephalic with normal ICP

Representative in vivo MRI scans show that the brain including the olfactory bulb of the p73^−/−^ mouse were strikingly smaller in comparison to the p73^+/+^ mouse (Fig. 1G, H, J and K). The statistical analysis confirmed that the brain tissue volume of the p73^−/−^ mice was significantly reduced by 25% compared to p73^+/+^ mice (p < 0.001, Table 3). In addition, the olfactory bulb of the p73^−/−^ mice was severely hypoplastic with a ~ 60% reduction in volume (Table 3). A prominent feature of the p73^+/+^ control mice was the appearance of very small cerebral ventricles (Fig. 1G, H), whereas in the ciliopathy p73^−/−^ mice, the cerebral ventricles were grossly enlarged (Fig. 1J, K and Table 3). The total cerebral ventricle volume, expressed as a fraction of TIV in p73^+/+^ and p73^−/−^ mice was 1.0% and 7.3%, respectively. The T1 maps revealed mild reflux of Gd-DOTA tracer into the 3rd ventricle of p73^−/−^ but not the p73^+/+^ mice (Fig. 1I and L). We measured ICP in separate series of p73^+/+^ and p73^−/−^ mice (Table 1). There were no ICP differences across the ciliopathy p73^−/−^ and p73^+/+^ control mice (p73^+/+^ (N = 4) ICP = 3.7 ± 1.1 mmHg vs p73^−/−^ (N = 5) ICP = 2.4 ± 0.6 mmHg, mean difference = 1.3 mmHg 95% CI [− 1.9, 4.5] mmHg, p-value = 0.325). Given observations of normal ICP and enlarged cerebral ventricles, we assigned the p73^−/−^ mice with normal pressure communicating type of hydrocephalus.

**Table 3:** Summary of baseline in vivo brain compartment volumes across p73^+/+^ control and p73^−/−^ groups

Dependent variable	p73^+/+^ (N = 8)		p73^−/−^ (N = 7)		Difference	SE	p-value	L95%	U95%
	Mean	SE	Mean	SE					
Total intracranial volume (TIV) mm^3^	453.3	6.3	362.3	6.6	91.0	9.1	**<** **0.001**	71.3	110.8
Brain parenchymal volume (mm^3^)	442.7	7.0	333.8	5.2	107.8	8.4	**<** **0.001**	89.5	125.9
Cerebral ventricle volume mm^3^	4.4	0.4	26.3	2.9	− 21.9	2.9	**<** **0.001**	− 29.0	− 14.7
CV volume fraction of TIV (%)	0.98	0.08	7.3	0.8	− 6.3	0.8	**<** **0.001**	− 8.3	− 4.3
Olfactory bulb volume (mm^3^)	19.2	0.5	7.7	0.5	11.5	0.7	**<** **0.001**	10.0	13.0

Data are presented as means and SE’s; Mean differences compare p73^+/+^ control vs. p73^−/−^ groups for each dependent variable using a two-sided independent samples t-test. L95%: lower limit of 95% confidence interval for mean (CI), U95%: upper limit of 95% CI

p-values marked with bold indicate that the mean difference is significant at the 0.05 level.

CV: Cerebral ventricle

The p73 protein is essential for brain development and previous studies reported abnormal hippocampal anatomy in p73^−/−^ mice [34]. The higher spatial resolution ex vivo scans confirmed hippocampal dysgenesis in the p73^−/−^ mice in comparison to control p73^+/+^ mice (Additional file 6: Fig. S4A, C, E, G). Specifically, the pyramidal CA1 cell layer appeared in a wave-like pattern and the granule cell layer of the dentate gyrus was severely underdeveloped (Additional file 6: Fig. S4C, G). In line with these observations, the hippocampus volume was significantly smaller in the p73^−/−^ in comparison to p73^+/+^ mice [p73^+/+^ (N = 8) hippocampus volume = 23.4 ± 0.4 mm^3^ vs p73^−/−^ (N = 6) hippocampus volume = 20.1 ± 0.6 mm^3^, mean difference = 3.7 mm^3^, 95% CI [2.2, 5.2] mm^3^, p-value < 0.001]. In addition, thinning of the cortex in the p73^−/−^ in comparison to p73^+/+^ control mice was also notable (Additional file 6: Fig. S4A–D). Compared to the FOXJ1-Cre;CEP164^fl/fl^, the choroid plexus of the p73^−/−^ mice appeared smaller (red arrows, Additional file 6: Fig. S4C, D and H). Indeed, the average volume of the choroid plexus within the lateral ventricles of the p73^−/−^ mice (N = 6) was 0.18 ± 0.02 mm^3^, which would translate into ~ 0.2 mg. The choroid plexus could not be quantified in the post-mortem specimens of the p73^+/+^ mice (Additional file 6: Fig. S4F). The observation of a smaller choroid plexus in p73^−/−^ mice compared to the FOXJ1-Cre;CEP164^fl/fl^ mice raise the possibility that CSF secretion differences might exist across the two ciliopathy mouse models and should be further explored in future in vivo studies. Clinically this is of particular interest since choroidal CSF hypersecretion has been associated with certain types of hydrocephalus [46, 47].

### Glymphatic transport is sustained in FOXJ1-Cre;CEP164^fl/fl^ and increased in p73^−/−^ mice

**Fig. 2:**
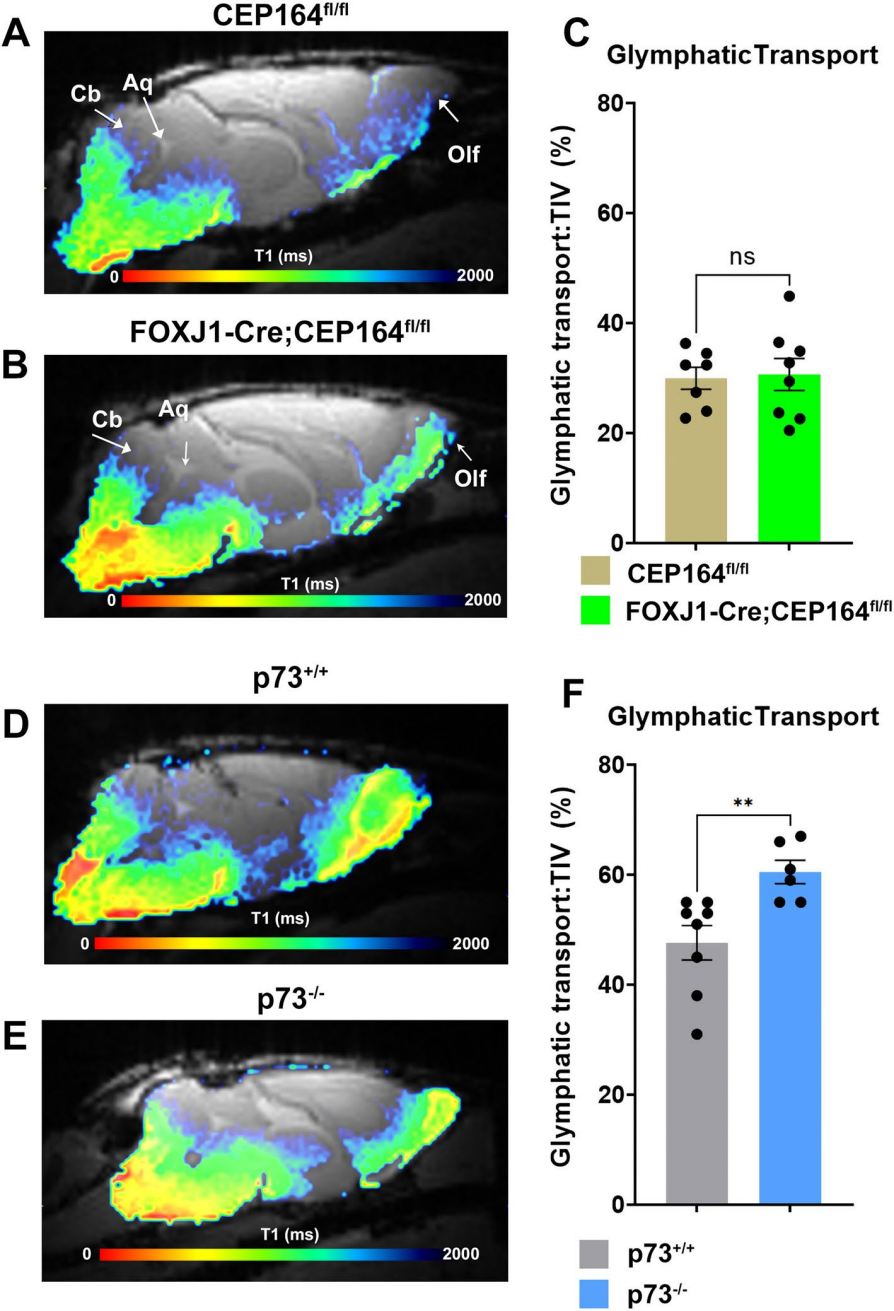
Glymphatic transport is sustained in FOXJ1-Cre;CEP164^fl/fl^ and increased in p73^−/−^ mice. **A**, **B** Representative color coded glymphatic transport T1 maps (brain parenchymal compartment) from a CEP164^fl/fl^ and a FOXJ1-Cre;CEP164^fl/fl^ mouse. Red and blue colors represent low and high T1 values, respectively. Note that low and high T1 values represent tissue areas with high and low glymphatic transport, respectively. **C** Graph with quantification of total glymphatic transport in % of total intracranial volume (TIV) across CEP164^fl/fl^ and FOXJ1-Cre;CEP164^fl/fl^ mice. Data are mean ± SEM. ns = no significant difference across the groups. **D**, **E** Color coded glymphatic transport maps (% of TIV) from a p73^+/+^ and a ciliopathy p73^−/−^ mice. **F** Graph with quantification of total glymphatic transport (% of TIV) between p73^+/+^ and p73^−/−^ mice. Data are mean ± SEM. **p-value = 0.006. Note the hypoplastic olfactory bulb in both ciliopathy mouse models. Olf: Olfactory bulb; cb: cerebellum; aq: aqueduct

To evaluate the effects of MCC dysfunction on CNS fluid homeostasis in the setting of hydrocephalus we measured glymphatic transport across the experimental groups using the quantitative T1 mapping technique [38]. With the T1 mapping technique Gd-DOTA is administered into the CSF via the CM and the glymphatic measurements are recorded ~ 1 h later [38]. T1 values in the range of 1–1700 ms represent transport of Gd-DOTA via the glymphatic system [38]. Brain maps color coded for glymphatic transport from a CEP164^fl/fl^ control mouse (Fig. 2A) and a FOXJ1-Cre;CEP164^fl/fl^ mouse (Fig. 2B) are shown, with red and blue colors representing high and low glymphatic transport, respectively. The glymphatic transport map from the CEP164^fl/fl^ shows that Gd-DOTA is distributed in a typical pattern for the rodent brain [38] with higher uptake in the cerebellum, brainstem, and olfactory bulb (Fig. 2A). Glymphatic transport in FOXJ1-Cre;CEP164^fl/fl^ mouse appears in a similar pattern to that observed in the CEP164^fl/fl^ control (Fig. 2B). The quantitative analysis confirmed that glymphatic transport was comparable across the two strains: (CEP164^fl/fl^ control (N = 7): 143.3 ± 10.0 mm^3^ vs FOXJ1-Cre;CEP164^fl/fl^ (N = 8): 156.2 ± 12.9 mm^3^, mean difference = − 12.7 mm^3^ 95% CI [− 48.1, 22.6] mm^3^, p-value = 0.449). Notably, when correcting for differences in TIV, the % of glymphatic tissue transport remained comparable as shown in Fig. 2C (CEP164^fl/fl^ control (N = 7): 30.0 ± 2.0% vs FOXJ1-Cre;CEP164^fl/fl^ (N = 8): 30.7 ± 3.0%, (p-value = 0.845). Glymphatic transport in p73^+/+^ and p73^−/−^ mice is shown in Fig. 2D and E, respectively. In the p73^+/+^ mouse, brain-wide glymphatic transport can be observed in a normal pattern (Fig. 2D). Glymphatic transport in the ciliopathy p73^−/−^ mouse was similarly vigorous and active in the cerebellum, brainstem, midbrain, forebrain, and olfactory bulb (Fig. 2E). The quantitative analysis confirmed that whole-brain glymphatic transport was comparable across the two strains: p73^+/+^ control (N = 8): 215.0 ± 12.7 mm^3^ vs p73^−/−^ (N = 6): 218.5 ± 7.1 mm^3^, mean difference = − 3.5 mm^3^ 95% CI [− 35.7, 28.7] mm^3^, p-value = 0.816. However, when correcting for differences in total brain volume, the quantitative analysis revealed that glymphatic transport was significantly increased by ~ 20% in the p73^−/−^ compared to controls as shown in Fig. 2F: (p73^+/+^ control (N = 8): 47.6 ± 3.1% vs p73^−/−^ (N = 6): 60.5 ± 2.1%, mean difference = − 12.9% 95% CI [− 21.2, 14.6] mm^3^, p-value = 0.006).

### Drainage via the cribriform plate to the nasal cavity is impaired in MCC ciliopathy mice

**Fig. 3:**
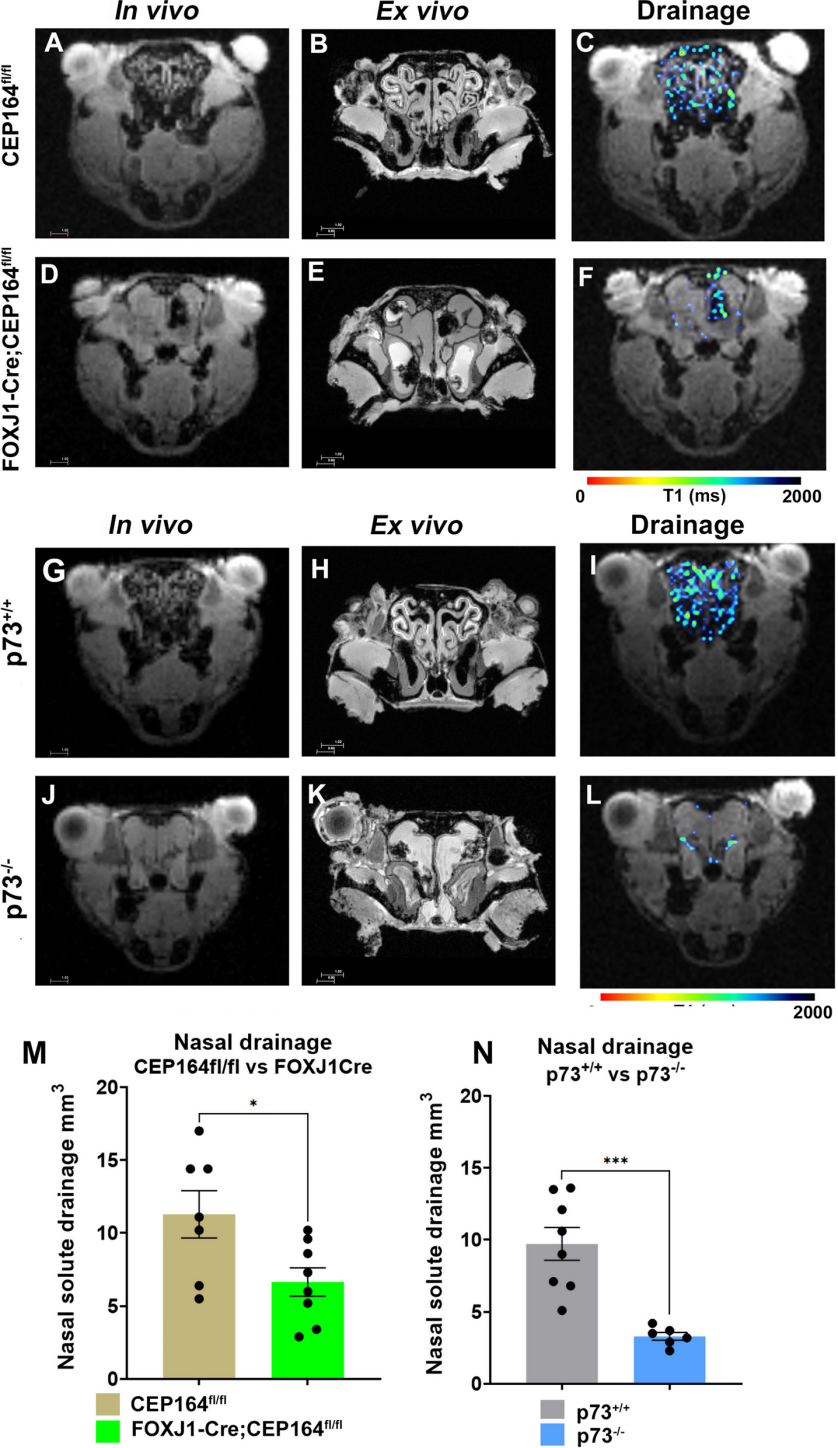
Drainage from the glymphatic system to the nasal cavity is reduced in ciliopathy. **A**, **B** In vivo and ex vivo anatomical axial MRI scans at the level of the nasal cavity from a CEP164^fl/fl^ mouse. **C** Drainage to the nasal cavity—measured as voxels in the range of 1–1700 ms from the T1 maps—displayed as a color-coded mask overlaid on the corresponding anatomical MRI from a CEP164fl/fl mouse. **D**, **E** In vivo and ex vivo anatomical MRI scans from a FOXJ1-Cre;CEP164^fl/fl^ mouse showing obliteration of the spiral lamellae and fluid filled maxillary recesses. **F** T1 map from the FOXJ1-Cre;CEP164^fl/fl^ mouse showing impaired drainage to the nasal cavity. **G**, **H** In vivo and ex vivo MRI scans at the level of the nasal cavity from a p73^+/+^ control mouse. **I** Corresponding T1 map at the level of the nasal cavity from a p73^+/+^ control mouse showing normal drainage pattern. **J**, **K** Anatomical nasal cavity MRI scans from a ciliopathy p73^−/−^ mouse showing abnormal anatomy and fluid filled maxillary recesses. **L** Corresponding T1 map from the ciliopathy p73^−/−^ mouse showing minimal drainage to the nasal cavity. **M** Graph with quantification of Gd-DOTA drainage to the nasal cavity across CEP164^fl/fl^ and FOXJ1-Cre;CEP164^fl/fl^ mice. Data are mean ± SEM. *p-value = 0.034. **N** Graph with quantification of Gd-DOTA drainage to the nasal cavity between p73^+/+^ and p73^−/−^ mice. Data are mean ± SEM. **p-value < 0.001

The observation of sustained or increased glymphatic transport in the two ciliopathies should be contextualized together with concurrent information on solute drainage from the CNS. The T1 mapping technique allows for parallel quantification of solute drainage through the cribriform plate to the nasal cavity as well as to the dcLN [38]. The MRI scans at the level of the nasal cavity from a CEP164^fl/fl^ mouse (Fig. 3A) showed the expected arrangement of the endoturbinates with spiral lamellae as well as the maxillary recesses in vivo as well as ex vivo (Fig. 3B). Corresponding in vivo (Fig. 3D) and ex vivo (Fig. 3E) scans from a FOXJ1-Cre;CEP164^fl/fl^ mouse revealed that the anatomy of the nasal cavity is strikingly different from that of the control CEP164^fl/fl^ mouse. In FOXJ1-Cre;CEP164^fl/fl^ mice, the endoturbinates were obliterated and filled with a substance characterized by homogeneous signal intensity likely representing accumulated mucus and infectious material associated with chronic rhinitis due to severe ciliopathy in the upper airways in this mutant [33]. Drainage of Gd-DOTA into the nasal cavity—as measured by voxels in the range of 1–1700 ms from the T1 maps—revealed a normal drainage pattern in the CEP164fl/fl mouse (Fig. 3C). However, the corresponding data from the FOXJ1-Cre;CEP164^fl/fl^ mouse (Fig. 3F) showed only a few voxels in the range of 1–1700 ms, signifying severely impaired drainage to the nasal cavity. The quantitative analysis confirmed that drainage to the nasal cavity was significantly reduced in the ciliopathy FOXJ1-Cre;CEP164^fl/fl^ mice as shown in Fig. 3M: (CEP164^fl/fl^ (N = 7): 11.3 ± 4.2 mm^3^ vs FOXJ1-Cre;CEP164^fl/fl^ (N = 8): 6.7 ± 2.7 mm^3^, mean difference = 4.6 mm^3^ 95% CI [0.4, 8.9] mm^3^, p-value = 0.034). For the p73 mice, the anatomical in vivo and ex vivo nasal cavity MRI and nasal drainage maps from the p73^+/+^ mice showed normal anatomy and a drainage pattern similar to the control CEP164^fl/fl^ mice (Fig. 3G–I). However, for the mutant p73^−/−^ mice, the MRI data revealed obliterated endoturbinates of the nasal conchae (Fig. 3J, K) and severely impaired Gd-DOTA drainage via the cribriform plate to nasal cavity (Fig. 3L). Figure 3N shows the significantly reduced drainage of Gd-DOTA to the nasal cavity in the p73^−/−^ mice compared to p73^+/+^: (p73^+/+^ (N = 8): 9.7 ± 1.1 mm^3^ vs p73^−/−^ (N = 6): 3.3 ± 0.1 mm^3^, mean difference = 6.4 mm^3^ 95% CI [3.7, 9.1] mm^3^, p-value < 0.001).

### Drainage to the deep cervical lymph nodes is sustained in both ciliopathy mouse models

**Fig. 4:**
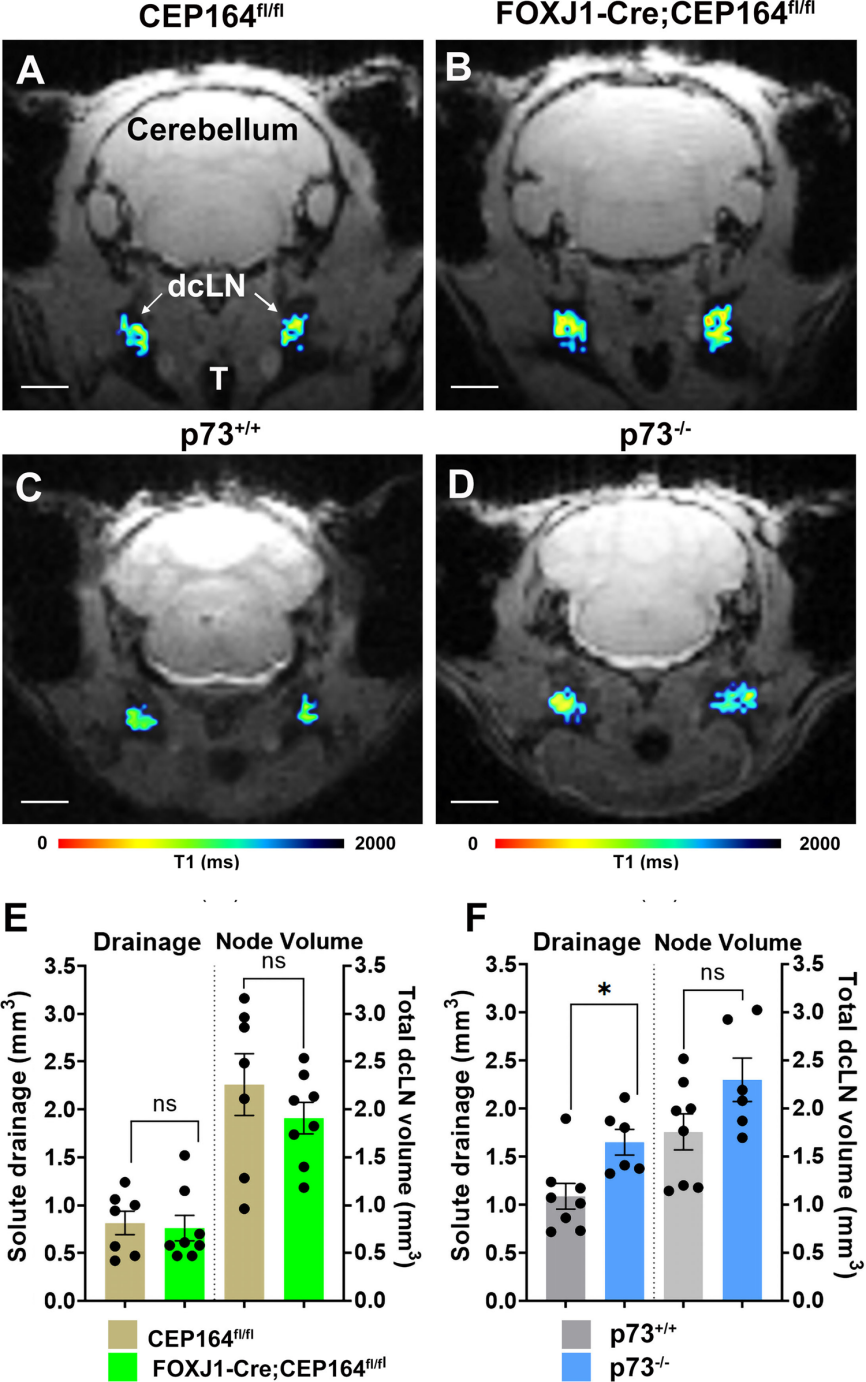
Drainage from CNS to the deep cervical lymph nodes is sustained in ciliopathy. **A**, **B** T1 maps of drainage to the deep cervical lymph nodes (dcLN) from a CEP164^fl/fl^ mouse and a ciliopathy FOXJ1-Cre;CEP164^fl/fl^ mouse. The drainage maps are color coded as T1 values in the range of 1–1700 ms, confined (masked) to the areas corresponding to the dcLN and overlaid onto the corresponding anatomical brain scans. Note that red and blue colors indicate low and high T1 values, respectively. **C**, **D** Corresponding T1 maps of drainage to the dcLN from a p73^+/+^ control mouse and a p73^−/−^ ciliopathy mouse. **E** Graphs with quantification of Gd-DOTA drainage to the dcLN (quantified as the volume of voxels with a T1 value in the range of 1–1700 ms) and the corresponding dcLN volumes across CEP164^fl/fl^ and FOXJ1-Cre;CEP164^fl/fl^ mice. Data are mean ± SEM. Graphs with quantification of Gd-DOTA drainage to the dcLN as well as the dcLN volumes across p73^+/+^ and p73^−/−^ mice. Data are mean ± SEM. *p-value = 0.012

Major waste disposal routes from the CNS include afferent lymphatics from the nasal cavity and the meningeal lymphatics that drain to the cervical lymph nodes, and in particular, the deep cervical lymph nodes (dcLN) [48, 49]. The T1 maps used for glymphatic mapping also permits evaluation of Gd-DOTA drainage to the dcLN in the same mice [38] and data was extracted across the groups. Figure 4 shows color-coded T1 maps of Gd-DOTA drainage to the dcLNs across the different mouse strains. The dcLNs are located posterolateral to the trachea and measure ~ 1 mm across. Drainage to the dcLN was measured as the number of voxels in the range of 1–1700 ms on the T1 maps and overlaid on the corresponding anatomical MRI scans (Fig. 4A–D). Gd-DOTA drainage to the dcLN in the ciliopathy FOXJ1-Cre;CEP164^fl/fl^ mice was similar to CEP164^fl/fl^ controls (Fig. 4A, B) and this was confirmed by the quantitative analysis across the groups shown in Fig. 4E: CEP164^fl/fl^ control (N = 7): 0.8 ± 0.1 mm^3^ vs FOXJ1-Cre;CEP164^fl/fl^ (N = 8): 0.75 ± 0.1 mm^3^, mean difference = 0.2 mm^3^ 95% CI [− 0.3, 0.4] mm^3^, p-value = 0.765). In contrast, the ciliopathy p73^−/−^ mice showed increased drainage to the dcLN when compared to controls (Fig. 4C, D). The quantitative analysis as shown in Fig. 4F confirmed increased drainage in the p73^−/−^ mice: (p73^+/+^ (N = 8) 1.1 ± 0.1 mm^3^ vs p73^−/−^ (N = 6) 1.7 ± 0.1 mm^3^, mean difference = − 0.6 mm^3^ 95% CI [− 1.0, − 0.2] mm^3^, p-value = 0.012). Because of the rhinitis (and persistent cough) observed in the ciliopathy strains we also measured the volume of the dcLN from the anatomical MRI to determine if the chronic infective state would cause lymphadenopathy and thereby affect the drainage results. As can be observed in Fig. 4E and F there were no differences noted in the total dcLN volume across the ciliopathy strains and their respective controls. Given that efflux via the cribriform plate to the nasal cavity was impaired in the two MCC ciliopathies, the finding of sustained solute drainage to the dcLN suggests that waste solutes rerouted to egress the CNS via other pathways to reach the dcLN.

### Peri-vascular AQP4 expression is altered in p73^−/−^ but not FOXJ1-Cre;CEP164^fl/fl^ mice

**Fig. 5:**
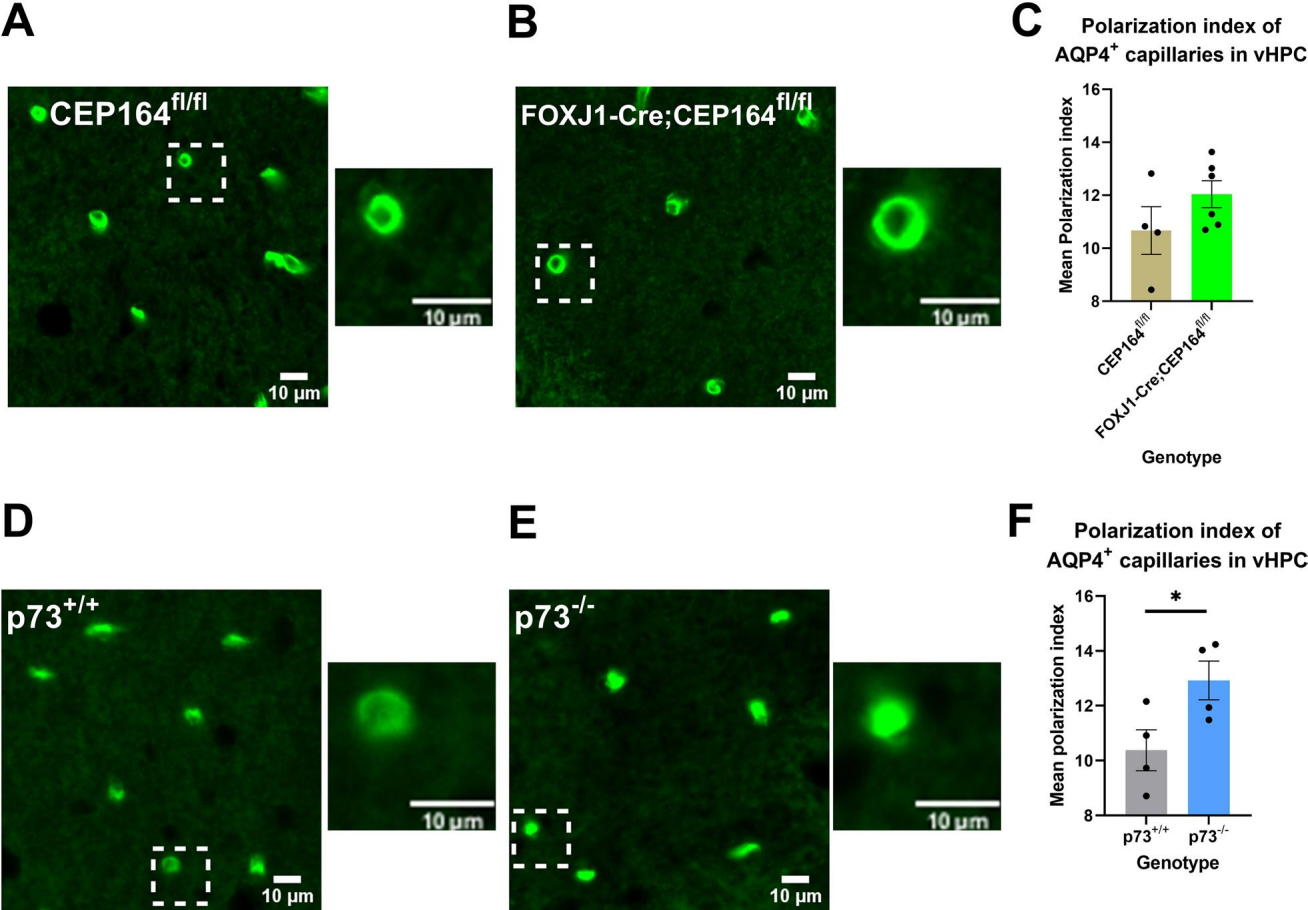
AQP4 expression is increased in p73^−/−^ but not in FOXJ1-Cre;CEP164^fl/fl^ mice. **A**, **B** Representative fluorescent micrographs of AQP4 expression in ventral hippocampus of p73^+/+^ mice and p73^−/−^ knockout mice. Image display settings are identical between micrographs in panels **A** and **B**. Subpanels of individual capillaries is magnification of dashed box in respective main image. **C** Mean polarization index of AQP4 around capillaries in ventral hippocampus was significantly increased in p73^−/−^ knockout mice relative to p73^+/+^ mice (p = 0.048). **D**, **E** Representative fluorescent micrographs of AQP4 expression in ventral hippocampus of CEP164^fl/fl^ mice and FOXJ1-Cre;CEP164 ^fl/fl^ knockout mice. Image display settings are identical between micrographs in panels **D** and **E**. Subpanels of individual capillaries is magnification of the dashed box in respective main image. **F** Mean polarization index of AQP4 around capillaries in ventral hippocampus did not significantly differ between CEP164^fl/fl^ and FOXJ1-Cre;CEP164^fl/fl^ knockout mice (p = 0.186). Scale bars = 10 μm. Mean value of all capillaries within an individual animal is represented indicated by black circle in panels **C** and **D**. Groupwise bars with error = Mean ± SEM. *p < 0.050

Since peri-vascular AQP4 water channel expression on the glial end-feet has been linked to glymphatic transport efficiency [50, 51], we conducted immunohistochemistry to characterize AQP4 expression across the strains. In the past, several reports of decreased peri-vascular AQP4 expression has been linked to neurodegenerative pathologies such as aging [10], cerebral small vessel disease [41] and Alzheimer’s disease [52]. In other words, when AQP4 is not localized to the glial end-feet but ‘dispersed’ elsewhere water mobility and glymphatic solute transport decreases. Notably, a recent study showed that the astrocytic end-feet on which periarterial AQP4 is expressed vary in size with the vasculature to optimize perivascular fluid pressure and perivascular-interstitial fluid exchange [53], and therefore we examined both capillaries (diameter < 10 µm) and small vessels (diameter ≥ 10 µm) for alterations in AQP4 polarization. There were no differences in the mean polarization index of AQP4 expression along small vessels and capillaries across FOXJ1-Cre;CEP164^fl/fl^ and CEP164^fl/fl^ mice (p-value = 0.240 and p-value = 0.155, respectively, Fig. 5A–C). In addition, for the FOXJ1-Cre;CEP164^fl/fl^ and CEP164^fl/fl^ mice there were no differences in mean radial CV of the AQP4 expression pattern of the microvasculature (Additional file 7: Fig. S5A, B) implying uniform circumferential AQP4 expression around the vessels. However, as shown in Fig. 5D–F the AQP4 expression pattern in the hippocampus was increased in the p73^−/−^ mice and confirmed by an increased AQP4 polarization index along the capillaries in p73^−/−^ mice in comparison to controls (p73^+/+^ (N = 4 mice, 431 to 998 capillaries analyzed per animal) mean polarization index 10.4 ± 0.7 a.u. vs p73^−/−^ (N = 4 mice, 408 to 878 capillaries analyzed per animal) mean polarization index 12.9 ± 0.7 a.u., mean difference = − 2.5 a.u. 95% CI [− 5.1, − 0.03] a.u., p-value = 0.048). Notably, there were also no differences in the mean radial CV of the AQP4 around the microvasculature in the p73^−/−^ and p73^+/+^ mice (Additional file 8: Fig. S6A, B) suggesting that the observed differences in the AQP4 polarization index (i.e., between p73^+/+^ and p73^−/−^) were not caused by highly localized clustering within each astrocytic endfoot or of a distinct subset of astrocytic endfeet, but rather supports the notion that AQP4 was upregulated uniformly along the entire perivascular aspect. Also, the peri-capillary AQP4 expression changes were unrelated to changes in capillary area fraction as shown in Additional file 8: Fig. S6C.

## Discussion

Here, we assessed glymphatic transport and solute drainage from the CNS in two different mouse models of ciliopathy. We used the MCC-specific CEP164 knockout (FOXJ1-Cre; CEP164^fl/fl^) and p73 knock-out (p73^−/−^) mouse models, both of which exhibit a significant loss of multicilia in the ependyma of the cerebral ventricles and communicating hydrocephalus. Our original hypothesis was that impaired ciliogenesis and severely reduced number of motile cilia in the MCCs in the cerebral ventricles would impair gross CSF flow through the CNS thereby decreasing glymphatic system transport and consequently solute waste drainage. This hypothesis was not corroborated as we observed that the glymphatic transport was sustained in FOXJ1-Cre; CEP164^fl/fl^ ciliopathy mice and was even increased in p73^−/−^ mice. However, in both ciliopathy models impaired solute drainage to the nasal cavity was striking and associated with a hypoplastic olfactory bulb and communicating hydrocephalus.

The finding of sustained and increased glymphatic transport in the two models of ciliopathy was unexpected given that these mice have defective MCCs, severe hydrocephalus and likely abnormal CSF flow. There are several mechanisms that can potentially explain our findings pertaining to glymphatic transport in ciliopathy. First, our data showed that the brain-wide distribution pattern of Gd-DOTA across the two ciliopathy models and their respective controls was comparable implying that glymphatic transport was normal in spite of ciliopathy. There are several known physiological drivers of glymphatic system transport including vascular pulsatility [16, 54], and vasomotion [17]. We tracked physiological variables during the MRI scans, and observed that heart rate was significantly increased in the p73^−/−^ compared to p73^+/+^ control mice (Additional file 3: Table S2), which may have contributed to the increased glymphatic transport observed in p73^−/−^ mice. Second, AQP4 water channels are important for peri-arterial glymphatic influx [50]. Our histological analyses revealed that, in comparison to respective controls, the capillary AQP4 polarization index in the ventral hippocampus was normal in FOXJ1-Cre; CEP164^fl/fl^ mice while increased in p73^−/−^ mice. Notably, the altered capillary AQP4 polarization of the p73^−/−^ mice was not confounded by changes in the microvascular area fraction. The increased capillary AQP4 polarization in the p73^−/−^ mice may potentially explain the increased glymphatic transport (measured in % of TIV). However, there is a gap in knowledge of how p73 might influence the expression of AQP4 and further experiments are required to understand the underlying mechanisms for the elevated peri-capillary levels of AQP4 in p73^−/−^ mice. Third, the sustained and increased glymphatic transport observed in the two ciliopathies must be considered together with drainage status for full interpretation. For any given static imaging approach designed to capture glymphatic system transport over a given study time, the presence of the solute of interest (e.g., Gd-DOTA or a fluorescently tagged tracers) in the brain parenchyma represents several dynamic processes: (1) influx from the subarachnoid space of Gd-DOTA dissolved in CSF via the peri-arterial conduits, (2) transport of Gd-DOTA from the perivascular conduits into the interstitial fluid space, (3) Gd-DOTA transport in the interstitial fluid towards egress routes which is a process dominated by diffusion [41] and (4) drainage of Gd-DOTA from the CNS. In the FOXJ1-Cre; CEP164^fl/fl^ and p73^−/−^ mice, solute drainage to the nasal cavity was severely impaired, creating a potential imbalance between solute influx and drainage. Thus, normal, or increased glymphatic transport observed in the ciliopathy mice might reflect longer transit passage times of Gd-DOTA through the glymphatic system due to impaired drainage along the olfactory cranial nerves. In rodents, a major drainage pathway for CSF is through the cribriform plate along the olfactory nerves and into the nasal cavity, which contains an extensive lymphatic network [55]. Further, alternative egress routes such as towards the spinal subarachnoid space as well as directly along other cranial nerve exits via the jugular foramen are also described. Finally, it is important to highlight that that although FoxJ1 is highly expressed in MCCs, it is also expressed in other tissues which might impacted the data in this particular strain. For example, FoxJ1 is expressed in the embryonic node, choroid plexus, and testis [56]. FoxJ1-Cre expression recapitulates expression of endogenous FoxJ1 [57]. Consistent with its expression in the embryonic node, it is required for establishment of the left–right body axis [58]. However, in FoxJ1-Cre;CEP164fl/fl mice, we have never seen randomization of the left–right axis, suggesting that either CEP164 is not required for formation of nodal cilia or Cre-mediated recombination is not efficient in the node. The latter is true in the testis. Even though FoxJ1-Cre is expressed in the testis, we did not see Cre-mediated recombination of CEP164 [59]. In terms of CNS, it has been shown that FoxJ1 is expressed in a small number of astrocytes [60] as well as temporarily in neuronal progenitor cells [61]. In summary, although it is unlikely, we cannot exclude the possibility that the CSF phenotypes may, in part, be attributable to cell types other than MCCs in FoxJ1-Cre;CEP164fl/fl mice.

The degree to which solute drainage from the glymphatic system into the nasal cavity under normal conditions is enabled (at least in part) by the cilia activity of the mucociliary epithelium remains unknown. However, the impaired drainage to the nasal cavity in the FOXJ1-Cre; CEP164^fl/fl^ and p73^−/−^ mice is more likely to be linked to the comorbidities associated with ciliopathy such as chronic rhinitis. The nasal cavity in both models of ciliopathy was abnormal secondary to the persistent purulent sinusitis and rhinitis known to afflict these animals [33, 34, 62]. Chronic infection is a result of MCC dysfunction in the respiratory epithelium of the p73^−/−^ and FOXJ1-Cre; CEP164^fl/fl^ mice and the inability to transport mucus. Ciliopathy mice are unable to clear nasal and tracheal secretions and suffer from chronic upper/lower respiratory tract infections, including cough, which likely impacts the lymphatic draining capacity via this route. Further, olfactory bulb hypoplasia observed in both ciliopathy models also plays a role. In particular, p73^−/−^ mice olfactory bulb hypoplasia is well documented [62], with p73 known to be essential for development and maintenance of neuronal numbers in the CNS, including the cortex and the olfactory bulb [63]. Global knockout of p73 implies that both TAp73 and ∆Np73 isoforms are missing [34]. The ∆Np73 isoforms are potent survival molecules for cortical and olfactory sensory neurons [63]. Pozniak et al., showed that in the absence of p73, the relative neuronal number in the cortex was decreased by ~ 35% and exhibited a dramatic loss of olfactory sensory neurons in the olfactory bulb [63]. The loss of olfactory sensory neurons, particularly in p73^−/−^ mice, is important from the point of view of impaired drainage to the nasal cavity because these neurons are critical for CSF/solute flow through the cribriform plate [64]. The olfactory axons act as a low resistance pathway for CSF and solute outflow from the CNS into the nasal cavity [65]. A recent study showed that axons of olfactory sensory neurons traverse the cribriform plate through small foramina in the cribriform plate in unison with blood vessels and lymphatic vasculature [64]. The effect of blocking CSF outflow through the cribriform plate on CNS fluid homeostasis has been investigated using a number of different approaches. In mice, chemical ablation of olfactory sensory neurons abolished the cribriform outflow pathway however, overall CNS fluid homeostasis appeared to adjust so that ICP did not increase [64]. Conversely, experiments in sheep with the cribriform plate sealed with bone wax resulted in ICP increases with controlled CSF infusions [66]. These studies imply that the nasal solute drainage route is an important factor for overall CNS fluid homeostasis, as well as waste solute clearance. It is important to highlight that our study showed that drainage of Gd-DOTA to the dcLN was sustained or increased in the ciliopathy mice. Several studies have mapped solute drainage from the CNS to cervical lymph nodes and report that the majority of waste disposal involves the dcLN, and less so the superficial lymph nodes [48, 49, 67]. Given that the nasal cavity lymphatics drain to the cervical lymph nodes, including the dcLN, we speculate that impairment of drainage to the nasal cavity in the ciliopathy mice resulted in rerouting of the anatomical egress pathways. Notably, Ahn et al. [68] has described the extensive meningeal lymphatics on the skull base which is in close proximity to the dcLN [13] and this network might become the major drainage pathway in the setting of impaired outflow to the nasal cavity.

Ciliopathies are often associated with neurological abnormalities [23, 25] and we also documented several brain aberrations in the FoxJ1-Cre;CEP164fl/fl and p73^−/−^ ciliopathy mice including hydrocephalus. The mechanism underlying development of communicating hydrocephalus in ciliopathy is still poorly understood. However, an increasing number of reports in mice, Xenopus and zebrafish have shown that the MCC (ependymal cells) lining the cerebral ventricles are important for: (i) CSF ‘near wall’ circulation [19, 20, 22], (ii) transport of nutrients and (iii) secretion of neuropeptides important for directional neural stem cell (NSC) migration (reviewed in Spassky and Meunier, 2017 [69]). Furthermore, genetic studies in humans with hydrocephalus and ciliopathy mouse models have uncovered defective neural stem cell proliferation including impaired cortical neurogenesis [60–62, 70]. Based on these studies a new model, known as the ‘NSC model’ of hydrocephalus, has emerged suggesting that impaired cortical neurogenesis (as observed in several mouse models of ciliopathy) inherently give rise to a ‘floppy’ cerebral cortex which is more compliant and therefore engender ventriculomegaly [71]. Accordingly, the hydrocephalus observed in the p73^−/−^ mice could be viewed in the context of a thinner and more ‘floppy’ cortex secondary to impaired cortical neurogenesis [63]. Nevertheless, while CSF volume replacement may indeed be part of the story, our new findings give rise to another distinct possibility that the hydrocephalic state in ciliopathy is more than passive and may also involve impaired CSF drainage via the nasal an observation which warrants future investigation.

## Conclusions

In summary, we showed that FOXJ1-Cre;CEP164^fl/fl^ mice presenting with the communicating form of hydrocephalus still demonstrated sustained glymphatic transport and AQP4 water channel expression along the microvasculature was normal when assessed in the ventral hippocampus. We also found that in p73^−/−^ mice glymphatic transport was even increased in comparison to p73^+/+^ controls, and this was paralleled by a significant increase in AQP4 polarization around capillaries. Drainage to the nasal cavity lymphatics via the cribriform plate was significantly reduced in both ciliopathy strains. To our knowledge, the combination of sustained glymphatic transport, impaired solute drainage via the cribriform plate to the nasal cavity and hydrocephalus has not previously been reported in vivo and may enhance our understanding of how different types of ciliopathies contribute to disruption of CNS fluid homeostasis, manifested in pathologies such as hydrocephalus.

## Supplementary Information

**Additional file 1: Figure S1.** Automated quantification of AQP4 polarization index using CellProfiler.

**Additional file 2: Table S1.** Number of analyzed AQP4^+^ vessels per animal following CellProfiler Analyst classification.

**Additional file 3: Table S2.** Physiological variables during MRI scanning.

**Additional file 4: Figure S2.** Varying degree of hydrocephalus in FOXJ1-Cre;CEP164^fl/fl^ mice.

**Additional file 5: Figure S3.** Ex vivo brain morphometry of CEP164^fl/fl^ and FOXJ1-Cre;CEP164^fl/fl^.

**Additional file 6: Figure S4.** Ex vivo brain morphometry of p73^+/+^ and p73^−/−^.

**Additional file 7: Figure S5.** AQP4 expression is unchanged in the vasculature of FOXJ1-Cre;CEP164^fl/fl^ mice.

**Additional file 8: Figure S6.** AQP4 expression is increased around capillaries of p73^−/−^ mice.

## Data Availability

The data sets from the current study can be made available upon reasonable request.
