# Supplementary material for: Sustained glymphatic transport and impaired drainage to the nasal cavity observed in multiciliated cell ciliopathies with hydrocephalus
Source: Fluids Barriers CNS. 2022 Mar 5;19:20. doi: 10.1186/s12987-022-00319-x (PMC8898469; doi:10.1186/s12987-022-00319-x)
Supplement: Supplementary file 1 — Additional file 1: Figure S1. Automated quantification of AQP4 polarization index using CellProfiler. [file 12987_2022_319_MOESM1_ESM.docx]

| **Additional File 1: Figure S1**.  **Automated quantification of AQP4 polarization index using CellProfiler** |
| --- |
| 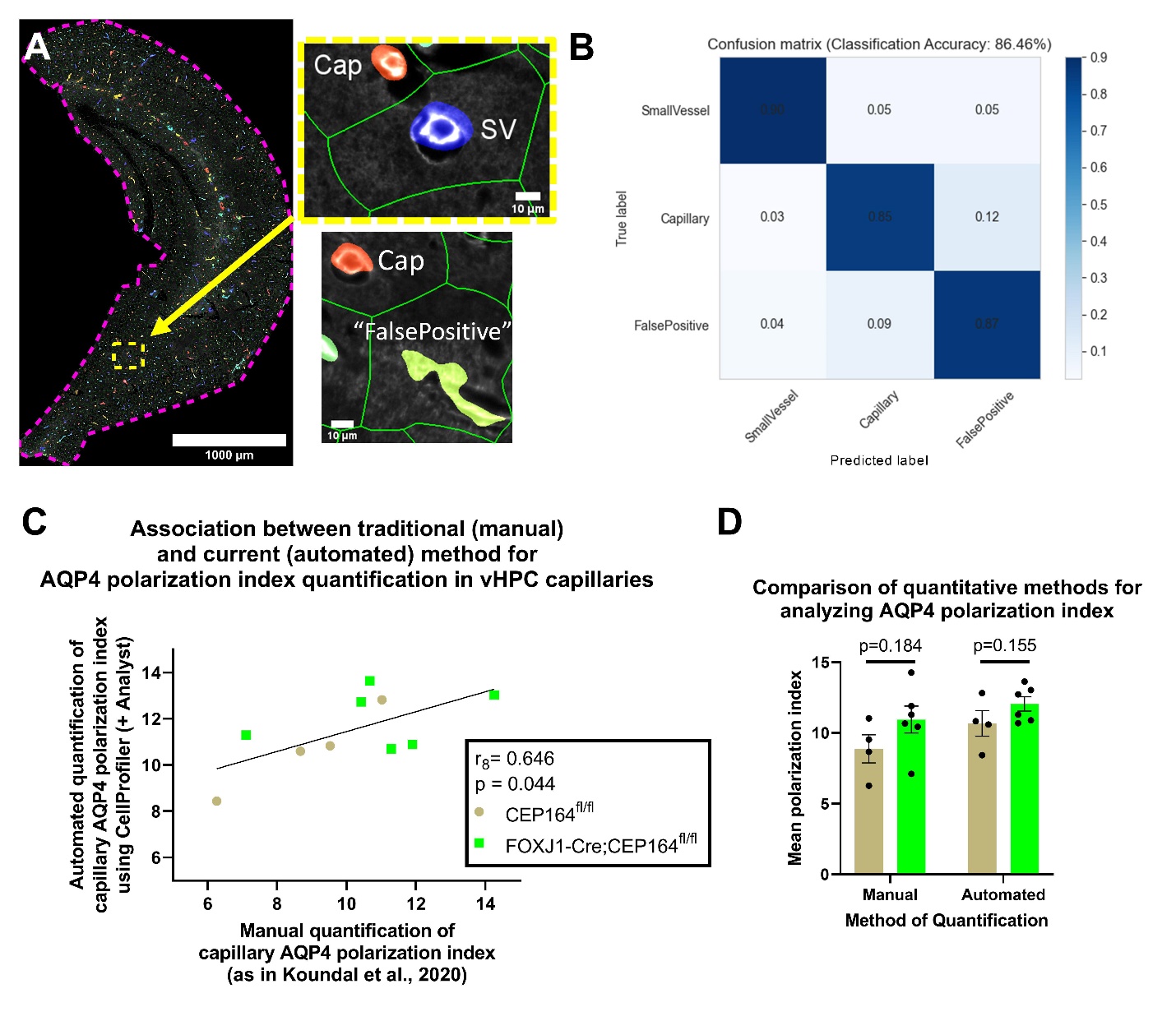 |
| **(A)** A representative image from vHPC with CellProfiler 4.1.3 object identifications overlaid. Subpanels on right demonstrate individual objects identified and their classification as either a capillary (“Cap”), a small vessel (“SV”), or neither (“FalsePositive”) using a neural network classifier in CellProfiler Analyst 3.0.2. Green contours represent the area around each object where the median value of background intensity was quantified for polarization index calculation. Text, yellow/magenta dotted lines, and scale bars are overlaid using FIJI/ImageJ for illustrative purposes and were not present in the CellProfiler quantification pipeline. **(B)** A descriptor of classifier accuracy, the “confusion matrix” output of the neural network classifier in CellProfiler Analyst 3.0.2 indicated that the neural network classifier had 86.46% accuracy. 201 randomly selected objects identified in the CellProfiler 4.1.3 pipeline were used as a training set. **(C)** The mean AQP4 polarization index of ventral hippocampal (vHPC) capillaries in CEP164fl/fl and FOXJ1-Cre;CEP164fl/fl animals was quantified using the previously published manual quantification technique to validate the use of the automated CellProfiler platform for this analysis. Briefly, the manual method consisted of random selection of 20 capillaries in the ventral hippocampus and calculation of polarization index using intensity values along a 100μm line that bisects each capillary. The polarization index of all 20 observations was averaged to get a single mean value per animal. The mean polarization index for each animal (represented by each data point) was correlated between the two techniques (r8=0.646, p=0.044), indicating that comparable data were obtained using the automated quantification platform. **(D)** Substantive interpretation of between-group analyses for mean AQP4 polarization index were identical regardless of quantification method used. There were no significant differences in AQP4 mean polarization index between the CEP164fl/fl (brown bars) and FOXJ1-Cre;CEP164fl/fl (green bars) genotypes using the previously published manual method nor the automated method in the current study. Groupwise bars with error = Mean ± SEM. |
