# Supplementary material for: Sustained glymphatic transport and impaired drainage to the nasal cavity observed in multiciliated cell ciliopathies with hydrocephalus
Source: Fluids Barriers CNS. 2022 Mar 5;19:20. doi: 10.1186/s12987-022-00319-x (PMC8898469; doi:10.1186/s12987-022-00319-x)
Supplement: Supplementary file 2 — Additional file 2: Table S1. Number of analyzed AQP4+ vessels per animal following CellProfiler Analyst classification. [file 12987_2022_319_MOESM2_ESM.docx]

**Additional File 2: Table S1**

Number of analyzed AQP4^+^ vessels per animal following CellProfiler Analyst classification

| Experiment | Genotype | Animal ID | # of  AQP4^+^ vessel observations | |
| --- | --- | --- | --- | --- |
|  |  |  | Capillaries | Small Vessels |
| p73  knockout | p73^+/+^ | A0740 | 431 | 47 |
|  |  | A0991 | 898 | 131 |
|  |  | A1199 | 998 | 223 |
|  |  | A1205 | 752 | 752 |
|  | p73^-/-^ | A0812 | 878 | 181 |
|  |  | A1136 | 851 | 263 |
|  |  | A1197 | 408 | 69 |
|  |  | A1209 | 781 | 259 |
| CEP164  knockout | CEP164^fl/fl^ | 07 | 754 | 134 |
|  |  | 3_3_HL | 562 | 46 |
|  |  | 5_2_HR | 885 | 140 |
|  |  | 9_2_HR | 947 | 342 |
|  | FOXJ1-Cre;  CEP164^fl/fl^ | 05 | 649 | 814 |
|  |  | 1_2_HR | 985 | 171 |
|  |  | 1_0_HR | 1042 | 269 |
|  |  | 1_2_HL | 947 | 341 |
|  |  | 65 | 1024 | 184 |
|  |  | 8_NH | 1028 | 222 |
