# Supplementary material for: Sustained glymphatic transport and impaired drainage to the nasal cavity observed in multiciliated cell ciliopathies with hydrocephalus
Source: Fluids Barriers CNS. 2022 Mar 5;19:20. doi: 10.1186/s12987-022-00319-x (PMC8898469; doi:10.1186/s12987-022-00319-x)
Supplement: Supplementary file 3 — Additional file 3: Table S2. Physiological variables during MRI scanning. [file 12987_2022_319_MOESM3_ESM.docx]

**Additional file 3: Table S2: Physiological variables during MRI scanning**

|  | **CEP164^fl/fl^**  **(N=10)** | | **FOXJ1Cre; CEP164^fl/fl^**  **(N=10)** | | **CEP164^fl/fl^** vs **FOXJ1Cre; CEP164^fl/fl^** | | | | |
| --- | --- | --- | --- | --- | --- | --- | --- | --- | --- |
| **Physiological variable** | **Mean** | **SE** | **Mean** | **SE** | **Difference** | **SE** | **L95%** | **U95%** | **P-value** |
| Respiratory rate (breath/min) | 154.7 | 7.5 | 167.5 | 5.1 | -12.8 | 9.1 | -32.1 | 6.5 | 0.179 |
| Heart rate (beats/min) | 213.7 | 8.1 | 229.3 | 13.0 | -15.6 | 15.3 | -49.5 | 18.3 | 0.331 |
| Body temperature (^o^C) | 36.6 | 0.1 | 36.6 | 0.1 | -0.03 | 0.2 | -0.4 | 0.3 | 0.850 |
| Body weight (g) | 24.4 | 0.8 | 22.9 | 0.3 | 1.6 | 0.9 | -0.4 | 3.5 | 0.110 |
|  | | | | | | | | | |
|  | | | | |  | | | | |
|  | **p73^+/+^ (N=8)** | | **p73^-/-^ (N=8)** | | **p73^+/+^ vs p73^-/-^** | | | | |
| **Physiological variable** | **Mean** | **SE** | **Mean** | **SE** | **Difference** | **SE** | **L95%** | **U95%** | **P-value** |
| Respiratory rate (breath/min) | 110.4 | 5.4 | 111.4 | 16.1 | -1.1 | 17.0 | -40.9 | 38.8 | 0.952 |
| Heart rate (beats/min) | 267.8 | 12.2 | 322.7 | 13.7 | -55.0 | 18.4 | -94.8 | -15.1 | **0.011** |
| Body temperature (^o^C) | 37.0 | 0.1 | 36.5 | 0.3 | 0.5 | 0.3 | -0.2 | 1.2 | 0.152 |
| Body Weight (g) | 22.9 | 1.4 | 18.8 | 0.8 | 4.0* | 1.6 | 0.54 | 7.5 | **0.027** |
