# Supplementary material for: Sustained glymphatic transport and impaired drainage to the nasal cavity observed in multiciliated cell ciliopathies with hydrocephalus
Source: Fluids Barriers CNS. 2022 Mar 5;19:20. doi: 10.1186/s12987-022-00319-x (PMC8898469; doi:10.1186/s12987-022-00319-x)
Supplement: Supplementary file 4 — Additional file 4: Figure S2. Varying degree of hydrocephalus in FOXJ1-Cre;CEP164fl/fl mice. [file 12987_2022_319_MOESM4_ESM.docx]

| **Additional File 4: Figure S2**.  **Examples of varying degree of hydrocephalus in FOXJ1-Cre;CEP164^fl/fl^ mice.**  **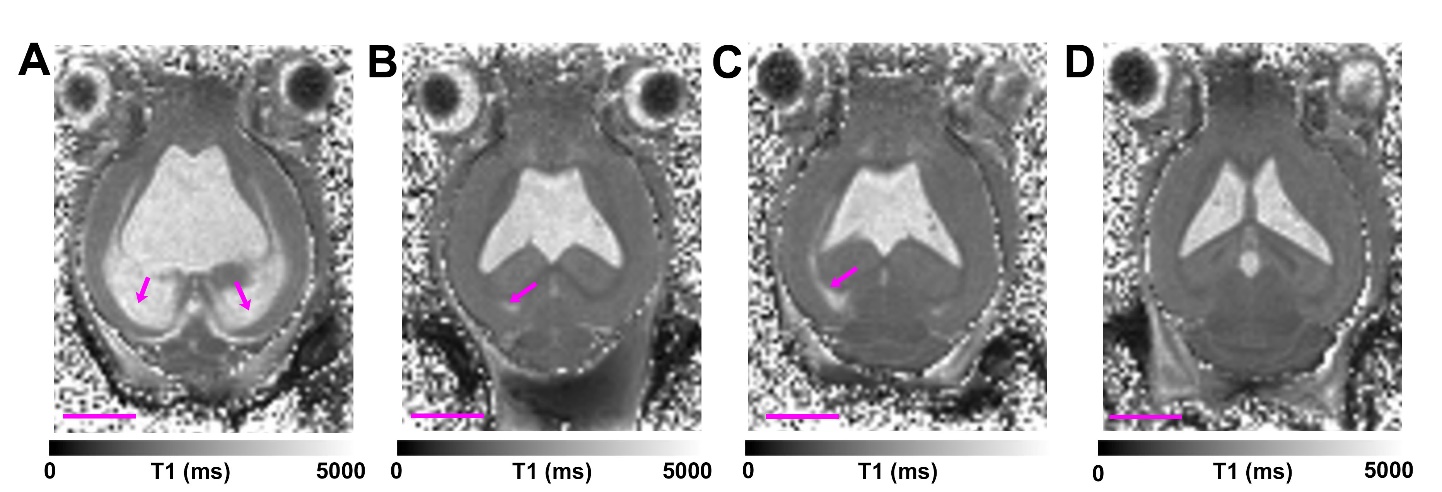** |
| --- |
|  |
| **A-D** T1 maps from four different FOXJ1-Cre;CEP164^fl/fl^ mice at the level of the lateral ventricles illustrating varying degree of enlarged ventricles as well as fluid tracking into the brain parenchyma (indicated by magenta colored arrows). Note that the FOXJ1-Cre;CEP164^fl/fl^ mouse in ‘**A’** has severe hydrocephalus and massive parenchymal fluid accumulation. The quantitative T1 maps was used to illustrate the enlarged ventricles as the fluid has high T1 values (~3-4000ms) and appears as a bright signal. Scale bars = 3mm. |
