# Supplementary material for: Sustained glymphatic transport and impaired drainage to the nasal cavity observed in multiciliated cell ciliopathies with hydrocephalus
Source: Fluids Barriers CNS. 2022 Mar 5;19:20. doi: 10.1186/s12987-022-00319-x (PMC8898469; doi:10.1186/s12987-022-00319-x)
Supplement: Supplementary file 5 — Additional file 5: Figure S3. Ex vivo brain morphometry of CEP164fl/fl and FOXJ1-Cre;CEP164fl/fl. [file 12987_2022_319_MOESM5_ESM.docx]

| **Additional file 5: Figure S3**  *Ex vivo* brain morphometry of CEP164^fl/fl^ and FOXJ1-Cre;CEP164^fl/fl^ |
| --- |
| 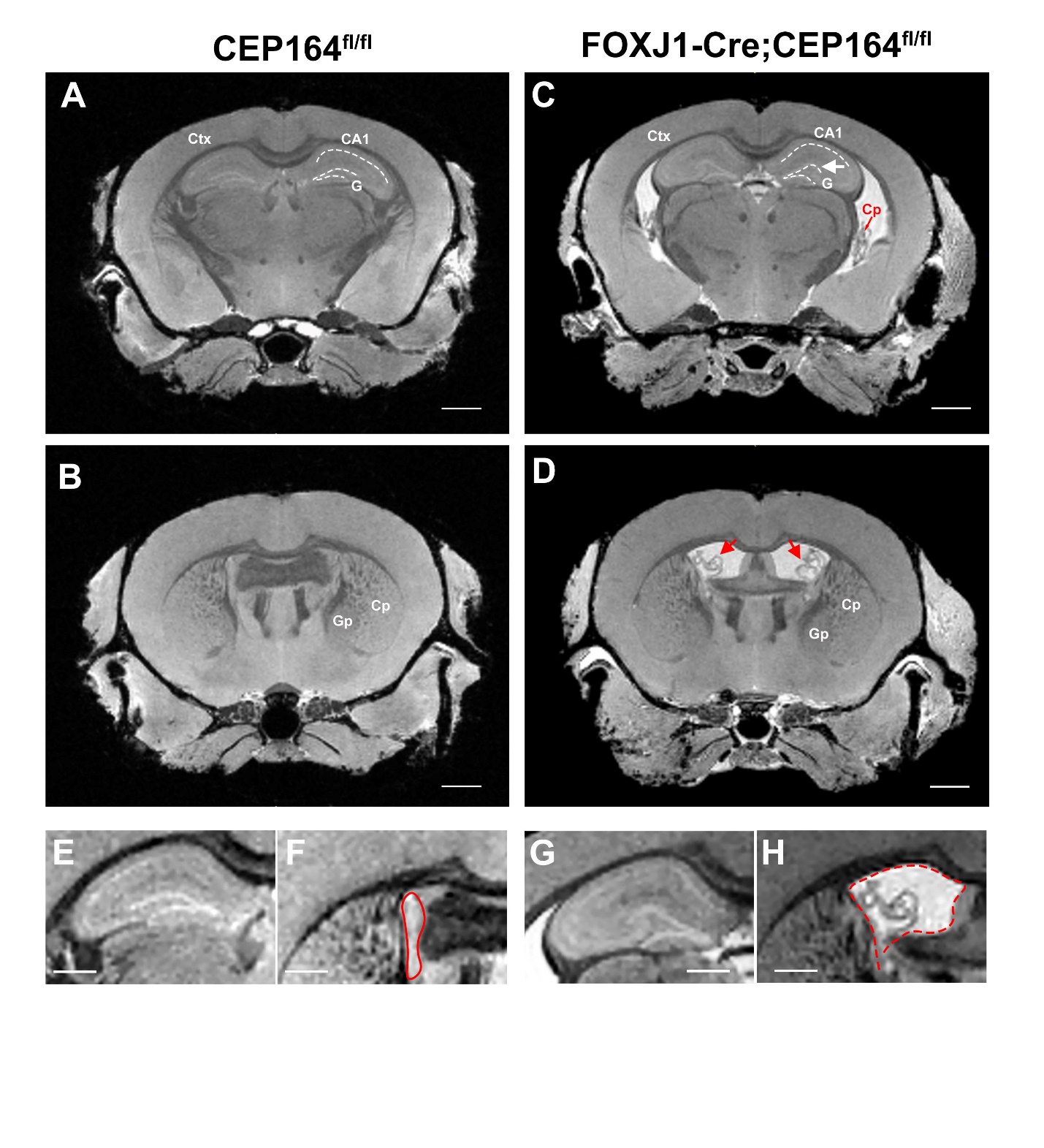 |
| *Ex vivo* MRI images from a control CEP164^fl/fl^ and a FOXJ1-Cre;CEP164^fl/fl^ mouse  level of the dorsal hippocampus (**A, C**) and striatum (**B, D**) are shown. White dashed lines indicate the pyramidal cells of the CA 1 and the granule cell layers of the dentate gyrus. White arrow points to a sharp bend in the granule cell layer. The choroid plexus (Cp) is visible in the lateral ventricles (red arrows). Ctx = cortex; G = granule cells; Gp = globus pallidus, Cp = caudate putamen. Scale bars = 3mm. **E, F** Higher magnification MRI images of the dorsal hippocampus and lateral ventricle from a CEP164^fl/fl^ mouse. The lateral ventricle is outlined in red. Scale bar = 1mm. **G, H** Corresponding MRI images from a FOXJ1-Cre;CEP164^fl/fl^ mouse. The lateral ventricle is outlined in red. Scale bars = 1mm. |
