# Supplementary material for: Sustained glymphatic transport and impaired drainage to the nasal cavity observed in multiciliated cell ciliopathies with hydrocephalus
Source: Fluids Barriers CNS. 2022 Mar 5;19:20. doi: 10.1186/s12987-022-00319-x (PMC8898469; doi:10.1186/s12987-022-00319-x)
Supplement: Supplementary file 6 — Additional file 6: Figure S4. Ex vivo brain morphometry of p73+/+ and p73−/−. [file 12987_2022_319_MOESM6_ESM.docx]

| **Additional file 6: Figure S4**  *Ex vivo* brain morphometry of p73^+/+^ and p73^-/-^ |
| --- |
| 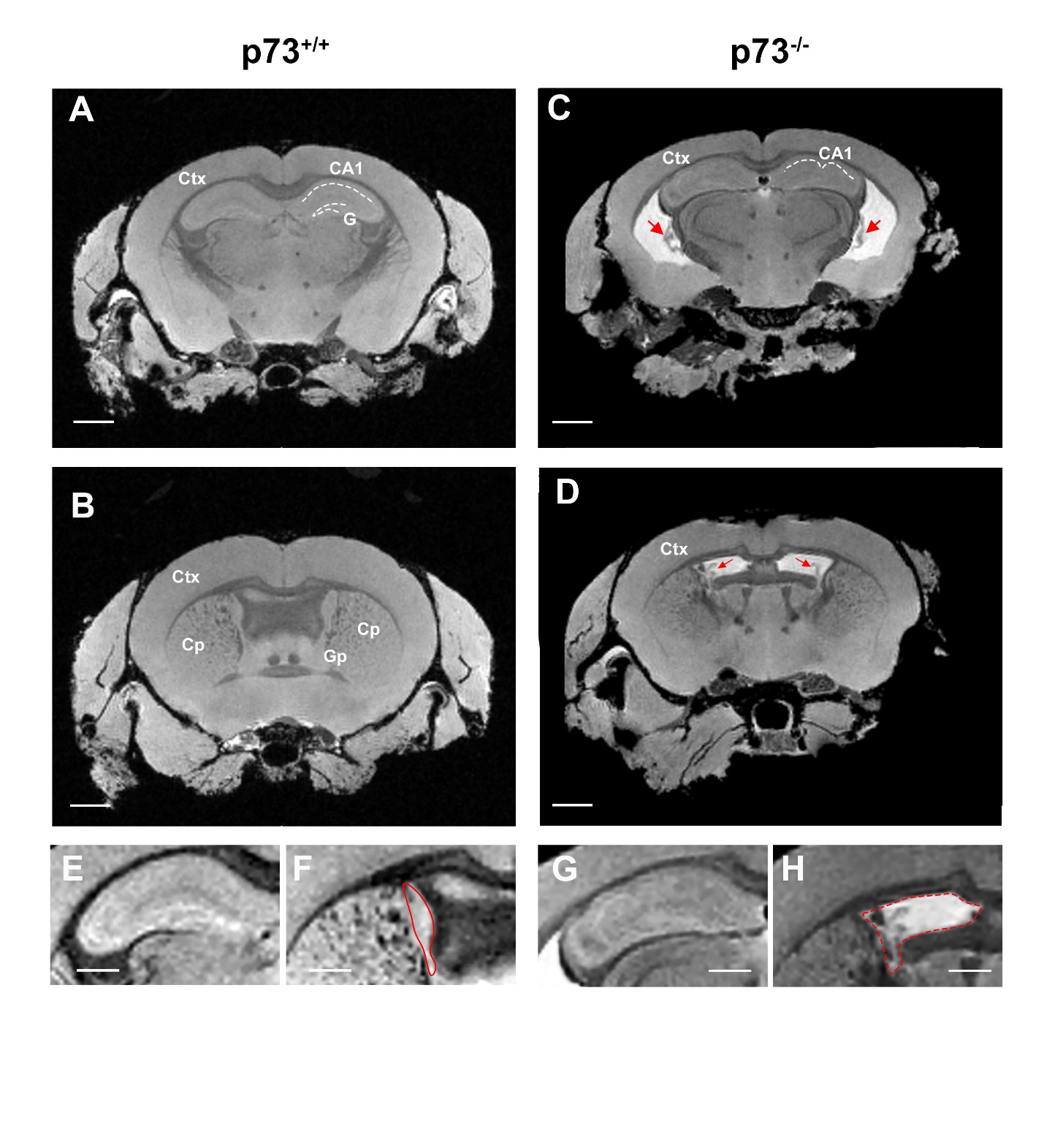 |
| *Ex vivo* MRI images from a control p73^+/+^ and a p73^-/-^ mouse at level of the dorsal hippocampus (**A, C**) and striatum (**B, D**) are shown. White dashed lines indicate the pyramidal cells of the CA1 and the granule cell layers of the dentate gyrus. Note that the CA1 pyramidal cell layer of the p73^-/-^ mouse appears as a wavy line. The choroid plexus (Cp) is visible in the lateral ventricles layer of the p73^-/-^ mouse (red arrows in C, D). Ctx = cortex; G = granule cells; Gp = globus pallidus, Cp = caudate putamen. Scale bars = 3mm. **E, F** higher magnification MRI images of the dorsal hippocampus and lateral ventricle from a p73^+/+^ mouse. The lateral ventricle outlined in red is tiny and collapsed in the post-mortem specimen. Scale bar = 1mm. **G, H** Corresponding MRI images from a p73^-/-^ mouse. The lateral ventricle is outlined in red. Scale bars = 1mm. |
