# Supplementary material for: Sustained glymphatic transport and impaired drainage to the nasal cavity observed in multiciliated cell ciliopathies with hydrocephalus
Source: Fluids Barriers CNS. 2022 Mar 5;19:20. doi: 10.1186/s12987-022-00319-x (PMC8898469; doi:10.1186/s12987-022-00319-x)
Supplement: Supplementary file 7 — Additional file 7: Figure S5. AQP4 expression is unchanged in the vasculature of FOXJ1-Cre;CEP164fl/fl mice. [file 12987_2022_319_MOESM7_ESM.docx]

| **Additional file 7: Figure S5**  AQP4 expression is unchanged in the vasculature of FOXJ1-Cre;CEP164^fl/fl^ mice |
| --- |
| 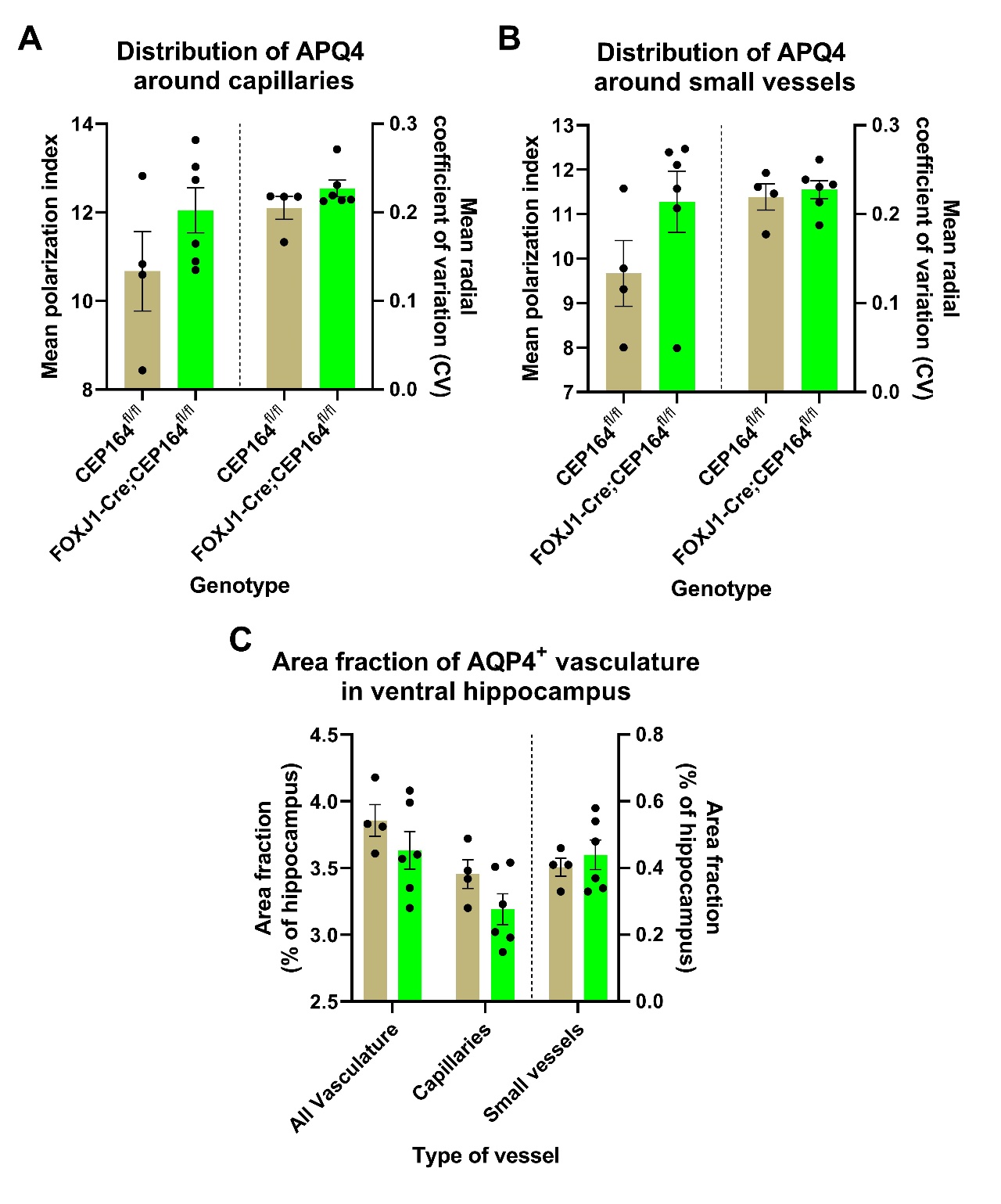 |
| **(A)** Mean polarization index and mean radial coefficient of variation (CV) of AQP4 around capillaries in ventral hippocampus did not significantly differ between CEP164^fl/fl^ and FOXJ1-Cre;CEP164^fl/fl^ mice. (**B)** Mean polarization index and mean radial CV of AQP4 around small vessels in ventral hippocampus did not significantly differ between CEP164^fl/fl^ and FOXJ1-Cre;CEP164^fl/fl^ mice. **(C)** The area fraction of AQP4+ vasculature was not significantly different between CEP164^fl/fl^ and FOXJ1-Cre;CEP164^fl/fl^ mice, either in total or when separated into capillary and small vessel subpopulations. Mean value of all capillaries or small vessels within an individual animal is represented by each black circle. Groupwise bars with error = Mean ± SEM. |
