# Supplementary material for: Sustained glymphatic transport and impaired drainage to the nasal cavity observed in multiciliated cell ciliopathies with hydrocephalus
Source: Fluids Barriers CNS. 2022 Mar 5;19:20. doi: 10.1186/s12987-022-00319-x (PMC8898469; doi:10.1186/s12987-022-00319-x)
Supplement: Supplementary file 8 — Additional file 8: Figure S6. AQP4 expression is increased around capillaries of p73−/− mice. [file 12987_2022_319_MOESM8_ESM.docx]

| **Additional file 8: Figure S6**  AQP4 expression is increased around capillaries of p73^-/-^ mice |
| --- |
| 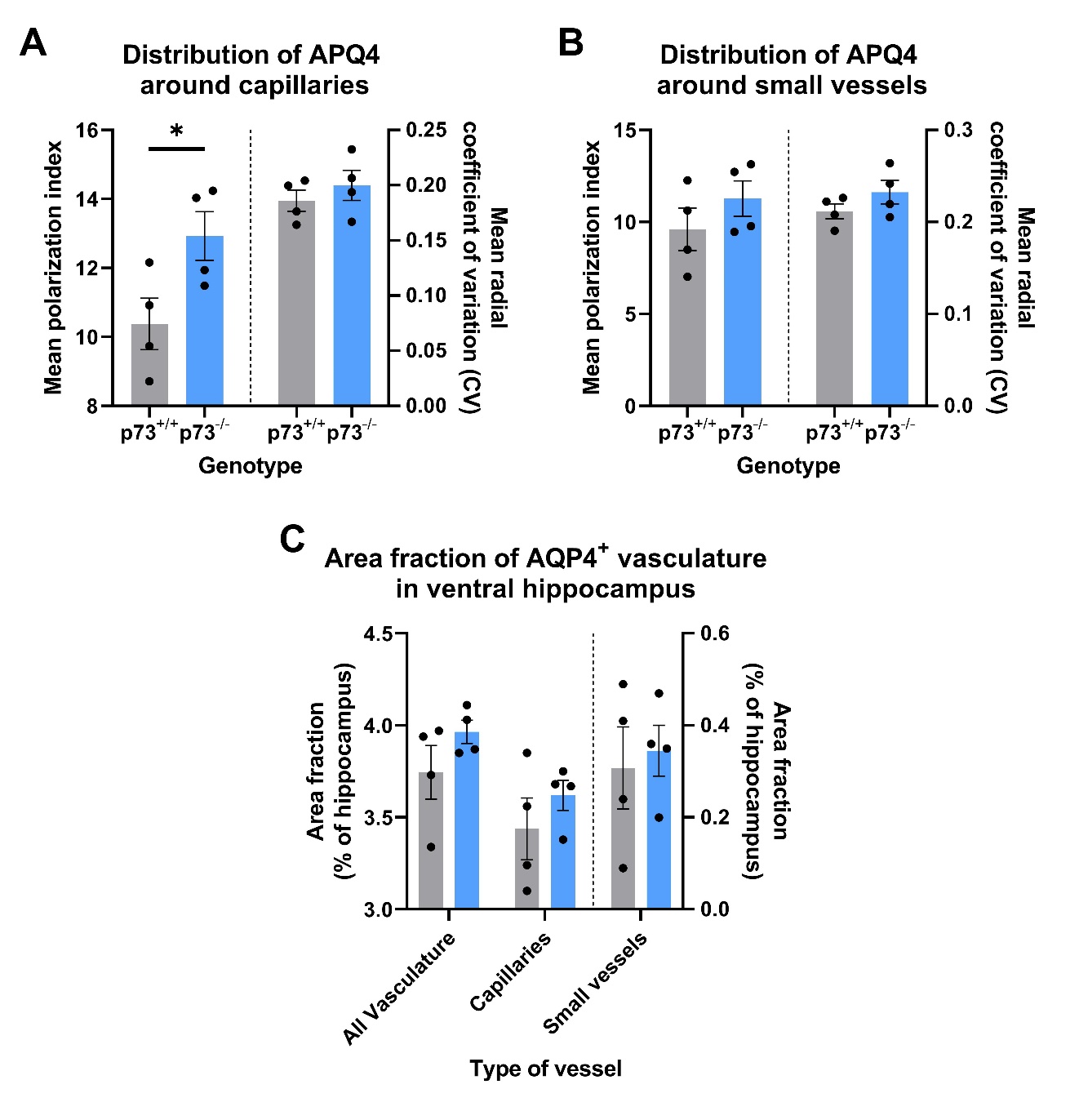 |
| **(A)** Mean polarization index of AQP4 around capillaries was significantly increased in p73^-/-^ knockout mice when compared with p73^+/+^ control animals. There was otherwise no significant difference in the distribution of AQP4 around capillaries as indicated by the mean radial coefficient of variation (CV) of AQP4+ signal around these capillaries. **(B)** There were no differences between p73^+/+^ and ^-/-^ genotypes in mean polarization index or mean radial CV of AQP4 expression around small vessels in ventral hippocampus. (**C)** The area fraction of the AQP4+ vasculature was not significantly different between p73^+/+^ and ^-/-^ mice, either in total or when separated into capillary and small vessel subpopulations. Mean value of all capillaries or small vessels within an individual animal is represented by each black circle. Groupwise bars with error = Mean ± SEM. * p<0.050. |
